# Marine-Derived Anticancer Agents Targeting Apoptotic Pathways: Exploring the Depths for Novel Cancer Therapies

**DOI:** 10.3390/md22030114

**Published:** 2024-02-28

**Authors:** Doralyn S. Dalisay, Chuckcris P. Tenebro, Edna M. Sabido, Angelica Faith L. Suarez, Melissa June V. Paderog, Rikka Reyes-Salarda, Jonel P. Saludes

**Affiliations:** 1 Center for Chemical Biology and Biotechnology (C2B2), University of San Agustin, Iloilo City 5000, Philippines; ctenebro@usa.edu.ph (C.P.T.); ednasabido@usa.edu.ph (E.M.S.); mpaderog@usa.edu.ph (M.J.V.P.); 2 Department of Biology, University of San Agustin, Iloilo City 5000, Philippines; rikka.salarda@isatu.edu.ph; 3 Balik Scientist Program, Department of Science and Technology, Philippine Council for Health Research and Development (DOST-PCHRD), Taguig 1631, Philippines; jsaludes@usa.edu.ph; 4 Center for Natural Drug Discovery and Development (CND3), University of San Agustin, Iloilo City 5000, Philippines; aicasuarez03@gmail.com; 5 Department of Pharmacy, University of San Agustin, Iloilo City 5000, Philippines; 6 Department of Chemistry, University of San Agustin, Iloilo City 5000, Philippines

**Keywords:** anticancer, marine sponges, apoptotic agents, marine actinobacteria, marine natural products, combination therapy, clinical trials, mechanism of action, marine natural products

## Abstract

Extensive research has been conducted on the isolation and study of bioactive compounds derived from marine sources. Several natural products have demonstrated potential as inducers of apoptosis and are currently under investigation in clinical trials. These marine-derived compounds selectively interact with extrinsic and intrinsic apoptotic pathways using a variety of molecular mechanisms, resulting in cell shrinkage, chromatin condensation, cytoplasmic blebs, apoptotic bodies, and phagocytosis by adjacent parenchymal cells, neoplastic cells, or macrophages. Numerous marine-derived compounds are currently undergoing rigorous examination for their potential application in cancer therapy. This review examines a total of 21 marine-derived compounds, along with their synthetic derivatives, sourced from marine organisms such as sponges, corals, tunicates, mollusks, ascidians, algae, cyanobacteria, fungi, and actinobacteria. These compounds are currently undergoing preclinical and clinical trials to evaluate their potential as apoptosis inducers for the treatment of different types of cancer. This review further examined the compound’s properties and mode of action, preclinical investigations, clinical trial studies on single or combination therapy, and the prospective development of marine-derived anticancer therapies.

## 1. Introduction

Apoptosis, also known as programmed cell death, refers to the physiological mechanism of “cell suicide” that occurs in a regulated manner during embryonic organogenesis and cellular differentiation [1,2]. The development and functioning of an organism are crucial for its overall well-being and survival [1,2]. Furthermore, it plays a critical role in the preservation of cell populations within tissues and serves as an immune defense mechanism against pathogen infections and the development of diseases through the activation of a cascade of caspases [1,3,4,5,6]. A diverse range of stimuli and conditions have been identified as catalysts for apoptosis, which can be classified into two main categories: intracellular and extracellular signaling. Intracellular signaling includes DNA damage, growth factor deprivation, and cytokine deprivation. On the other hand, extracellular signaling involves the production of death-inducing signals by cytotoxic T cells as part of the immune response [1,6]. In the p53 pathway, for instance, when intrinsic or extrinsic stress signals cause possible damage in the normal process of the cell cycle (in G1 and G2 stages), the p53 protein is activated in response to the stress signal. The activation of the protein induces either cell cycle arrest, known as cell senescence, or cellular apoptosis as a protective mechanism against potential harm that could lead to abnormalities in DNA replication and compromise the stability of the genome [7,8].

Dysregulation of apoptosis can give rise to a spectrum of pathological conditions, encompassing neurodegenerative disorders, ischemic injury, autoimmune ailments, and diverse forms of malignancy [1,9]. In cancer, for instance, the lack of the p53 gene or its functional protein confers a predisposition to early-onset cancer in organisms [9]. Malignant cells possess the capacity for metastasis and exhibit resistance to anticancer medications during the later stages [10,11]. The pursuit of targeting apoptotic pathways has emerged as a compelling approach in the quest for the advancement of chemotherapeutic agents [11,12,13].

Ongoing scientific investigations are dedicated to the exploration of compounds that selectively modulate cell death pathways distinct from caspase-dependent intrinsic apoptosis. These alternative pathways, such as autophagic cell death and mitotic catastrophe, hold potential for the development of innovative therapeutic approaches for cancer. Marine-derived natural products cover a vast reservoir of unique compounds that possess the potential to function as apoptotic agents. The structural classes of these compounds encompass a spectrum ranging from basic linear peptides, such as spisulosine [14], to complex fused tetrahydroisoquinoline, exemplified by trabectedin [15]. Marine organisms, including sponges, mollusks, tunicates, and other marine microorganisms, have been identified as significant contributors to the production of compounds that exhibit potential anticancer properties [16,17,18].

Marine-derived compounds can target apoptotic pathways in cancer cells by inducing cell death in multiple ways, such as by targeting cell cycle arrest [18], interfering with DNA [19] and mediating mitochondrial interactions [20]). In the case of didemnin, it exhibits a binding affinity towards the minor groove of DNA, which impedes the activity of transcription factors and DNA repair mechanisms. Consequently, this disruption leads to the activation of apoptosis [19]. In contrast, lamellarin D induces apoptosis through pore formation in the inner mitochondrial membrane, resulting in the activation of caspases and the subsequent initiation of apoptosis [20].

Throughout the course of time, a multitude of bioactive compounds derived from marine sources have been isolated and subjected to rigorous research. Nevertheless, a limited number of compounds have demonstrated effectiveness as inducers of apoptosis and have progressed to the stage of being tested in clinical trials [17,19]. These compounds are derived from a variety of organisms and exert their effects through multiple molecular mechanisms, targeting numerous pathways of apoptosis. Among the first marine-derived compounds approved for cancer treatment (sarcoma and ovarian cancer) is trabectedin [21,22]. It was initially isolated from a marine ascidian (*Ecteinascidia turbunata*); however, synthetic trabectedin is already available. Moreover, a synthetic version of trabectedin has since been developed and is now commercially accessible. Notably, this compound impedes trans-activated transcription and interact with DNA repair proteins involved in the synthesis of cytokines, chemokines, and anti-tumor agents [23].

At present, it is observed that a significant proportion of the anticancer medications available on the market, specifically 60%, are derived from natural sources [24]. A number of these compounds are sourced from marine resources and are currently undergoing clinical trials for their potential use in treating cancer [16,17,18,25]. This review provides a summary of 21 natural products obtained from marine sources that are presently studied in preclinical and clinical trials, along with their synthetic derivatives. These investigations aim to target the apoptotic pathway in various cancer types. The compounds are so chemically diverse that there is no common pharmacophore sharing one target in the apoptotic pathway. Thus, the compounds in this review are organized based on where they originated from, i.e., sponges, corals, tunicates, mollusks, ascidians, algae, cyanobacteria, fungi, and actinobacteria, rather than based on their chemical classification. This review article additionally discussed the identification of these compounds, their chemical characteristics and mechanism of action, preclinical studies, and the evaluation of the clinical trials conducted to assess their effectiveness either as single or combination therapies. Finally, this review offers a prospective analysis of the current state of research and development concerning anticancer drugs sourced from marine organisms.

## 2. Marine Sponges

### 2.1. Cytarabine (Ara-C)

**Figure 1:**
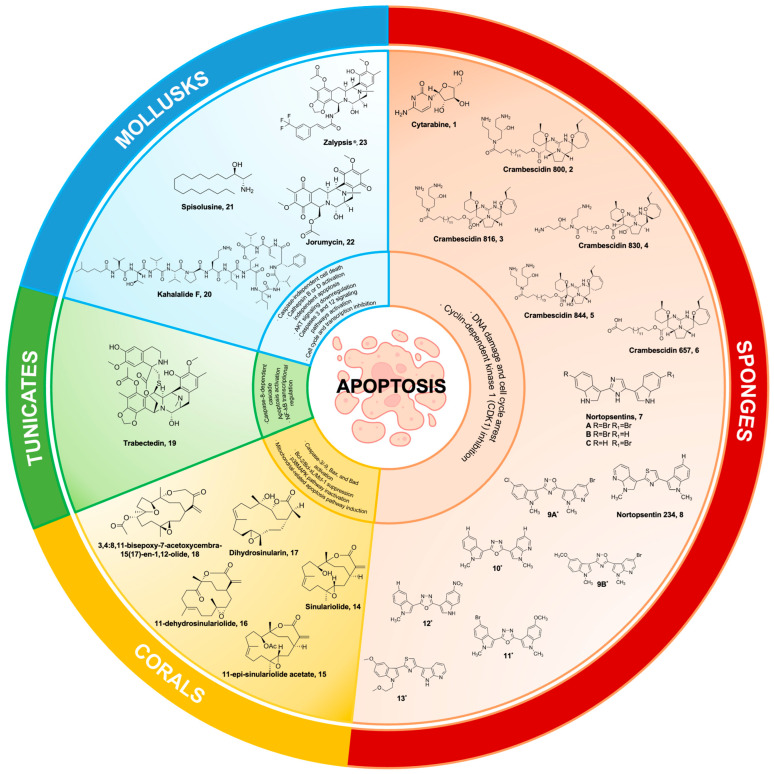
Marine natural products from sponges, corals, tunicates, and mollusks and their synthetic derivatives, with their mechanisms of action targeting the apoptosis pathways. 9A* 5-(5-bromo-1-methyl-1Hpyrrolo[2,3-b]pyridin-3-yl)-3-(5-chloro-1-methyl-1H-indol3-yl)-1,2,4-oxadiazole; 9B* 5-(5-bromo-1-methyl-1Hpyrrolo[2,3-b]pyridin-3-yl)-3-(5-methoxy-1-methyl-1Hindol-3-yl)-1,2,4-oxadiazole; 10* 2-(1-methyl-1H-indol-3-yl)-5-(1-methyl-1H-pyrrolo[2,3-b]pyridin-3-yl)-1,3,4-oxadiazole; 11* 2-(5-bromo-1-methyl-1Hindol-3-yl)-5-(5-methoxy-1-methyl-1H-indol-3-yl)-1,3,4-oxadiazole; 12* 2-(1-methyl-1H-indol-3-yl)-5-(5-nitro-1H-indol-3-yl)-1,3,4-oxadiazole; 13* 2-(5-methoxy-1-(2-methoxyethyl)-1H-indol-3-yl)-4-(1H-pyrrolo[2,3-b]pyridin-3-yl)thiazole.

For over sixty years, extensive research and development efforts have been dedicated to investigating the properties and applications of cytarabine **1** (Figure 1). Compound **1** is a synthetic derivative of C-nucleosides that was originally extracted from the marine sponge *Cryptotethya crypta*. Its primary focus has been on establishing its efficacy as a standard treatment option for individuals diagnosed with leukemia and lymphoma. Bergmann and Feeney [26,27] reported in the 1950s that these nucleosides and their derivatives possess a carbohydrate moiety that is covalently linked to either a purine or pyrimidine base. The arabinosyl nucleosides, which have been postulated to serve as a defense mechanism in sponges as they are predominantly found in an unbound state [27,28], have attracted the attention of researchers owing to their distinctive chemical characteristics and potential as anticancer agents. Hence, the available scientific information has a pivotal role in stimulating the development of numerous synthetic ara-nucleosides. To highlight, the first reported synthesis of **1** by the group of Walwick in 1959 has been an exceptional lead for drug development for cell apoptosis [29]. Compound **1** exerts its inhibitory effects on DNA synthesis through intracellular metabolism, specifically by converting into its active form, known as cytosine arabinose triphosphate. This active form competes with the natural substrate of DNA polymerase, deoxycytidine triphosphate, thereby impeding the synthesis of DNA [30,31]. As the cells divide rapidly, **1** acts at the S phase of the cell and hinders the progression of cells from the G1 phase to the S phase and, thus, reduces cell replication [32,33,34].

In 1969, pharmaceuticals containing **1** approved by the Food and Drug Administration (FDA) were accessible in two forms. The first form was conventional **1**, marketed under the brand name Cytosar-U^®^, which was prescribed for the treatment of acute lymphocytic leukemia, acute myelocytic leukemia, chronic myelogenous leukemia, and meningeal leukemia [35,36]. The second form was cytarabine formulated in liposomes, known as Depocyt^®^, which was specifically indicated for the intrathecal treatment of lymphomatous meningitis [37,38].

Despite the advancements in therapeutic outcomes achieved through the administration of **1** to individuals diagnosed with leukemia and other hematological malignancies, certain patients exhibit unfavorable prognoses because of acquired drug resistance. Consequently, the development of prodrugs and the implementation of combinatorial treatment strategies have become imperative. The efficacy of a combination therapy involving **1** and anthracycline (bacterial-derived anticancer) has been extensively assessed in various clinical investigations, encompassing acute leukemia [39,40], non-Hodgkin’s lymphoma [41], and acute promyelocytic leukemia [42]. For instance, CPX-351 is a drug composed of **1** and daunorubicin in 5:1 ratio. This drug demonstrated synergistic effects against P388 murine lymphocytic leukemia, with a combination index (CI) of less than 0.9. The drug combination exhibited additive effects against HL-60 cells, with CI ranging from 0.9 to 1.1 [43].

Moreover, one study has documented that the co-administration of **1** with idarubicin, an anthracycline chemotherapeutic agent, and indisulam, a sulphonamide compound, has exhibited superior efficacy in Phase II clinical trials involving patients with relapsed or refractory acute myeloid leukemia (AML) and high-risk myeloid syndrome characterized by the presence of damaged DNA-binding protein 1 (DDB1) and human cullin 4 (CUL4)-associated factor 15. Notably, this combination therapy achieved a notable response rate of 35% [44]. The co-administration of drugs with cytarabine showed no competitive inhibition of the adenosine triphosphate (ATP) binding site of cyclin-dependent kinase (CDK) enzymes. Consequently, these drugs hold promise as potential candidates for combination therapy with cell cycle-active agents and ATP-competitive cyclin-dependent kinase (CDK) inhibitors [45,46].

**Figure 2:**
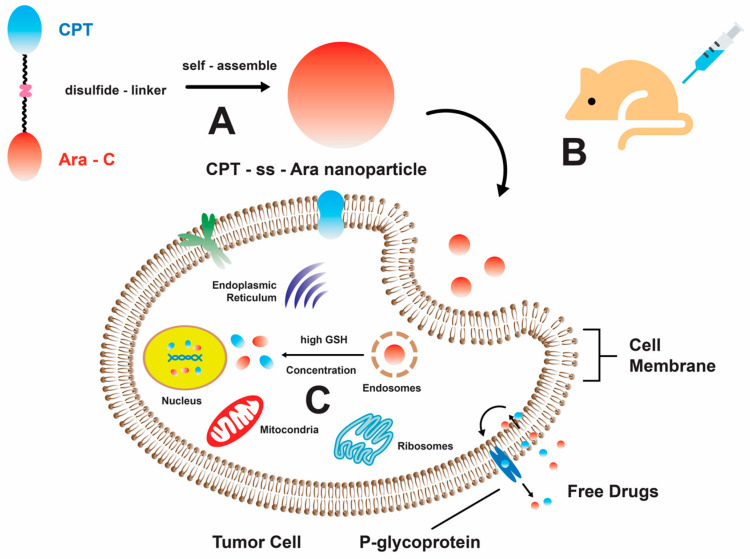
A schematic representation illustrating the self-assembly mechanism of the CPT-ss-Ara conjugate, which exhibits promising prospects in the field of cancer treatment. (**A**) The amphiphilic CPT-ss-Ara conjugates demonstrate self-assembly characteristics, resulting in the formation of nanoparticles upon introduction to an aqueous solution. (**B**) The neoplastic cells uptake nanoparticles via the mechanism of endocytosis. (**C**) The liberation of pharmaceutical compounds takes place via the disulfide bond cleavage process, which is initiated by glutathione (GSH) [55]. Reproduced with permission from W. He and X. Hu et al., “Rational Design of a New Self-Codelivery System from Redox-Sensitive Camptothecin-Cytarabine Conjugate Assembly for Effectively Synergistic Anticancer Therapy”, published by John Wiley and Sons, 2017.

Furthermore, the combination of quinolone-derived topoisomerase II inhibitors and **1** resulted in a synergistic effect and enhanced cellular apoptosis. According to the report, the synergistic activity between voreloxin, a derivative of quinolone, and **1** showed a CI value of less than 0.85. The IC50 of voreloxin against two human acute leukemia cell lines (MV4-11 and HL-60) were less than 95 nM and 884 nM, respectively [47]. Additionally, the administration of voreloxin significantly reduced reversible platelets and 89% bone marrow cellularity [47]. Cytarabine-induced DNA damage caused cells to transition from the S phase to the G2 phase [48], while voreloxin induced double-strand breaks in DNA, leading to cell death [49]. A Phase Ib/II study reported vosaroxin, a quinolone derivative drug that intercalates DNA and inhibits topoisomerase II [50,51,52,53]. A Phase III trial for this combination therapy was initiated with relapsed/refractory acute myeloid leukemia [49,50,54]. Another combination therapy was demonstrated by an alkaloid, camptothecin (CPT), that is chemically linked to **1** using a disulfide bond that can be cleaved by glutathione (GSH). This conjugation results in the formation of a drug–drug conjugate that is sensitive to changes in redox conditions. Through this mechanism, the substance can undergo self-assembly, resulting in the formation of nanoparticles. This process leads to enhancements in the water solubility and permeability of **1** across cell membranes (Figure 2) [55]. Upon investigating the combinatorial treatments, it was observed that most of these therapeutic agents are derived from planar, cyclic, and ring structures. These structural attributes are widely regarded as crucial factors in the inhibition of topoisomerases, as evidenced by previous studies [56,57,58].

Aside from chemical modification and combinatorial treatment with other nature-derived drugs, the introduction of potentiating groups such as small-noncoding RNA molecules is another strategy used to address problems on drug insensitivity and resistance [59,60,61,62,63]. One of the reports revealed that AMO-miR-21 in combination with cytarabine enhanced HL-60 cells’ apoptosis (IC50 < 1 μM), suggesting that the miR significantly sensitized HL-60 cells due to the upregulation of the programmed cell death 4 (PDCD4) gene [62]. Furthermore, another experiment was conducted to overexpress miR-134 in K562/A02 and HL-60/ADM cells [63]. This manipulation was performed with the aim of making these cells more responsive to **1**. The outcome of this intervention was a reduction in the formation of cell colonies and an enhancement in the ability of **1** to induce apoptosis. Cell apoptosis was induced by inhibiting the eukaryotic initiation factor 4E and downregulating the expression of myeloid cell leukemia-1 (Mcl-1) and B-cell lymphoma 2 (bcl2). The Mnks (mitogen-activated protein kinase (MAPK)-interacting kinases) gene was hypothesized to be a potential target of miR-134, a microRNA molecule [63]. This relationship was found to be inversely correlated with the expression levels observed in human multidrug-resistant (MDR) leukemia cells and samples from patients with relapsed or refractory acute myeloid leukemia (AML) [63].

The effectiveness of **1**, in conjunction with other pharmaceutical agents, in treating leukemia and other hematological malignancies has been well-documented. However, research has also demonstrated its ability to induce apoptosis, in various other cancer types, including breast cancer (specifically MDA MB-468 cells with an IC50 value of 10 μM) [64] and lung cancer [65,66,67]. In another findings, compound **1** have demonstrated that Y-family trans-lesion synthesis (TLS) DNA polymerase η (pol η) effectively overcomes the obstacle of DNA replication by efficiently extending from Ara-C-terminated primers [68]. The research findings indicate that Pol-η can accommodate **1** at various stages of the catalytic cycle. Furthermore, it has been observed that Pol-η can influence the conformation of **1** sugar molecule through specific interactions involving hydrogen bonding and stacking [68].

Treatments for AML may be improved by combining **1** with other anticancer agents, such as the protopanaxatriol metabolite K (CK). In AML cell lines, the hybrid **1**/CK therapy showed encouraging apoptotic induction and DNA damage [69]. In monocytic leukemia cell lines (THP-1 and U937), the results showed an increased ratio of deoxycytidine kinase/cytidine deaminase (dCK/CDA) gene expression, which further enhanced **1**-induced DNA damage [69]. The key challenge to cytarabine therapy and chemotherapeutic approaches continued to be the compound **1** resistance of AML cells. Reducing the expression of the G2/M checkpoint kinase Wee-1 (Wee1) in AML cells is an important target mechanism to mitigate cancer resistance and DNA repair [70]. Adavosertib and other Wee1 inhibitors functioned as the main source of interference for the delayed G2/M transition, which in turn caused the induction of apoptosis [70]. Interestingly, in the Jurkat acute lymphoblastic leukemia (ALL) animal system, the therapeutic combination of **1** and adavosertib changed important metabolic pathways [70]. The primary enriched processes, such as the mitochondrial electron transport chain, gluconeogenesis, and Krebs cycle, were disrupted with this combinatorial pharmacological treatment [70]. Collectively, the synergistic pharmacological interaction of **1** with adavosertib provided a more mechanistic understanding of **1**’s impact on all cell apoptosis, proliferation, and cancer development [70]. Even with the previously described antiAML characteristics, cytarabine administration in adult fruit flies was found to cause intestinal cell damage in a recent study [71]. This was mainly due to the upregulation of pro-apoptotic genes (reaper) and caspase genes (drice and dcp-1) [71]. In addition, **1** enhanced oxidative stress and proliferation in intestinal stem cells (ICSs) [71]. Compound **1**’s potential as an effective anticancer treatment exhibit encouraging prospects for the future. Advanced structural designs and drug delivery strategies hold promise for **1** in mitigating the challenges associated with an unfavorable prognosis and drug resistance.

### 2.2. Crambescidin and Its Derivatives

Marine polycyclic guanidine alkaloids (MGAs), crambescidin 800 **2**, crambescidin 816 **3**, crambescidin 830 **4**, crambescidin 844 **5** (Figure 1), and their derivatives were isolated from the Mediterranean sponge *Crambe crambe* [72,73]. Initial findings reported that these compounds were active against *Herpes simplex* virus, type 1 (HSV-1), and L1210 murine leukemia cells, with **3** being the most abundant and active component [73]. In addition, **3** was found to be active against HCT116 human colon carcinoma cells with an IC50 of 0.24 μg/mL [72]. Its cytotoxic activity was further tested on animal models and revealed that it can inhibit the reaggregation of *Ephydatia fluviatilis* cells as well as exhibit toxicity against the fish *Lethrinus reticulatus* (<50 mg/L) and the shrimp *Artemia salina* [72]. Its potency against human cancer cell lines and its total synthesis of complex molecular structure gave several groups the interest to further synthesize crambescidins [74,75,76,77,78,79]. Crambescidin 359 was successfully synthesized as pentacyclic guanidine units with spermidine or hydroxyspermidine as anchor units [79]. This was isolated together with other guanidine alkaloid dehydrobetzelladine C and crambescidin 431, which was toxic against nauplii of *A. salina* [80].

Over the recent decade, research investigations pertaining to crambescidin have predominantly centered on its capacity to inhibit HIV-1 [81,82,83]. The resumption of synthesizing side-chain analogs of crambescidin alkaloids occurred in 2004 as a response to the diminishing rate of the discovery of anticancer pharmaceuticals. It was observed that analogs of crambescidins 657 **6** and 800 **2** with increasing lengths of side chains exhibited promising anticancer activity against murine and human cancer cell lines [74]. Furthermore, it has been documented that **2** exhibits the ability to halt the progression of the S phase in K562 chronic myelogenous leukemia (CML) cells while simultaneously prompting their differentiation into erythroblasts [83]. In addition, a study investigated the cytotoxic activity of **6** and **2** against A431 human epidermoid carcinoma cells. The results revealed that these compounds exhibited significantly higher cytotoxicity, with an IC50 value of less than 10 μM. In contrast, analogs possessing shorter side chains demonstrated lower cytotoxic activity. This observation suggests that the extended aliphatic chain of the guanidine alkaloid plays a crucial role in modulating its permeability into animal cells [84]. Furthermore, it was observed that **2** exhibited the capability to stimulate cellular proliferation in breast cancer cells by causing a halt in the G2/M phase. This effect was achieved through the suppression of phosphorylation in the Akt, NF-kB, and MAPK signaling pathways [85]. Compound **2** reduced cellular proliferation in breast cancer cells by causing a halt in the G2/M phase. This effect was achieved through the suppression of phosphorylation in the Akt, NF-kB, and MAPK signaling pathways [86,87]. Monanchoxymycalin C (MomC), an isomeric analogue of **2**, demonstrated pro-apoptotic activity against HeLa cervical cancer cells by inducing cell cycle arrest at the S phase [88]. Moreover, the combined treatment of this pentacyclic guanidine alkaloid and cisplatin inhibited HeLa cancer cell growth and proliferation [88].

Additional research was conducted to investigate the potential anticancer properties of crambescidin. Specifically, **3** was examined for its ability to interact with Cav-1 or L-type calcium channels. This interaction resulted in the inhibition of voltage-gated calcium channels within neuroblastoma X glioma cell lines [86]. A report highlighted the cytotoxic impact of C816 on cortical neurons, resulting in reduced neuronal viability and a dose-dependent elevation of cytosolic calcium levels, particularly in younger neurons [87]. The investigations into the potential activity of **3** yielded results that extended beyond the inhibition of neuron channels [72,86,87]. Notably, **3** also exhibited potent activity against *Saccharomyces cerevisiae*, with an IC50 value of 4 μM after 24 h. This effect was achieved by inducing cell cycle arrest at the G2/M phase, subsequently leading to an augmentation in cell DNA content and size [89]. Furthermore, **3** exhibits cytotoxic effects on HT-29 colon carcinoma cells at a concentration of 150 nM after 24 h of incubation. Additionally, it demonstrates inhibitory effects on HepG2 cells by disrupting the G0/G1 phase of the cell cycle and reducing the expression of cyclins and cyclin-dependent kinases [89]. An in vivo assay was conducted to further investigate the effects of **3** in the zebrafish xenotransplantation model and the results indicated that the treated embryos exhibited a minor regression of tumors at 0.5 μM [90].

Overall, studies conducted on the apoptotic activity of crambescidins have brought attention to **2** and **3** as the primary active analogs of marine guanidine alkaloids (MGAs). These findings indicate that the bioactivities of these compounds are primarily influenced by their pharmacological structure. The compounds **2** and **3**, exhibit identical structural characteristics. They possess elongated aliphatic side chains that only vary at the C-13 position, where the presence of a hydroxyl group is observed in **3**. Significant structural variations have been documented, as well as variations in the specific cell lines that these compounds interact with. These findings have provided valuable insights for understanding the complex interaction of various polycyclic guanidine alkaloids with human cancer cells.

### 2.3. Nortopsentins

Nortopsentins **7** (Figure 1) are a type of bis-indolyl natural product derived from sea sponge *Spongosorites ruetzleri* [91]. They feature a distinct chemical scaffold with an imidazole positioned between their two indole units [92]. This makes them a promising source of anticancer chemicals when used against a murine leukemia cell line (P388) [91]. Imidazole and bis-indole scaffolds from **7** have undergone structural changes to maximize cytotoxic action and reduce adverse effects [93,94,95]. For example, nortopsentin 234 **8** is an analogue of **7** in which a thiazole and a 7-azaindole moiety, respectively, have been substituted for the core imidazole ring and one indole unit [96]. Cyclin-dependent kinase 1 (CDK1) was inhibited by the neo-synthetic bis(indolyl)thiazole analogue of **7** [96], which, when overexpressed, may promote uncontrolled tumor cell proliferation [97]. This derivative initially reduced cell proliferation and clonogenicity in colorectal cancer sphere cells (CR-CSphCs), but prolonged **8** treatment may result in an adaptive response in CR-CSphCs [98]. Following the treatment of **8**, the tiny subset of spared CR-CSphCs overexpressed the membrane receptor CD44 variant 6 (CD44v6), which was strongly linked to the wingless/integrate (Wnt) pathway’s activation [98]. These regrowth and resistance mechanisms of CR-CSphC may suggest an upregulation of checkpoint kinase 1 (CHK1) for enhanced CR-CSphC proliferation [98]. Interestingly, the synergistic effect of **8** and the CHK1-inhibitor rabusertib disrupted the Wnt pathway and induced apoptosis in both CD44v6-negative and CD44v6-positive colorectal cancer stem cell (CR-CSC) fractions [98]. The combinatorial treatment of **8** analogue with rabusertib displayed antiproliferative and anticlonogenic activity against CR-CSCs [98].

Reduced cell growth was seen in the MCF-7 breast cancer cell when 1,2,4-oxadiazole was substituted for the aromatic bis-indole linker found in **7** [99]. Notably, a 1,2,4-oxadiazole derivative **9** promoted G0/G1 cell cycle arrest in MCF-7 cancer cells [99]. Additionally, the 5-bromo-1-methyl-1H-pyrrolo[2,3-b]pyridine moiety in **9** may inhibit the Caco-2 colon cancer cell line’s ability to proliferate. In MCF-7 cancer cells, the synthesized 1,2,4-oxadiazole derivative caused nuclear condensation and blebbing in the cell membrane to cause early pro-apoptosis [99]. Meanwhile, the 1,3,4-oxadiazole ring substituent of **7** revealed a promising reduction of cell proliferation in a panel of pancreatic ductal adenocarcinoma (PDAC), including a primary PDAC culture and a gemcitabine-resistant variant [100]. With an IC50 value of 1.4 μM against PDAC cells, a specific derivative of this heterocyclic alteration, number **10**, demonstrated impressive anticancer activity [100]. Additionally, compound **10** was found to be an inhibitor of CDK1 (cyclin-dependent kinase 1) [101]. In a separate study, two other 1,3,4-oxadiazole derivatives (**11** and **12**) had IC50 values of 1.8 and 2.6 μM against MCF-7 cancer cells [102]. Additionally, compound **12** inhibited tumor cell proliferation in the lung (A549) cancer cell line at 3.3 μM, while anti-tumor potential was observed against the cervical (HeLa) cancer cell line at 6.34 μM.

In a dose-dependent manner, **7**’s napththyl-substituted indole induced pro-apoptotic and non-necrotic actions against MCF-7 cancer cells [102]. When the thiazole motif of a **7** analogue was modified with a 2-methoxyethyl group, the derivatives reduced the cell proliferation of MCF-7 cancer cells [103]. In particular, a very specific antiproliferative activity was shown by the thiazole derivative **13**, which was synthesized and had 5-methoxy and 2-methyloxyethyl components. It stopped the cell cycle in MCF-7 cancer cells during the G2/M phase [103]. Moreover, the IC50 of CDK1-inhibitor **13** was comparable to known CDK1 inhibitors [103]. Despite the lack of clinical trials for **7** and its derivatives, attempts are being made to find new chemical scaffolds developed from this anticancer compound obtained from marine sponges.

## 3. Marine Corals

### Sinulariolide

Sinulariolide **14** (Figure 1) is a cembranolide compound derived from the soft coral *Sinularia flexibilis*, which was collected in Maluku, Indonesia. The compound was extracted using methylene chloride following the removal of fats using hexane. Its structure was elucidated by Tursch in 1975 [104]. Due to its complex stereochemistry, the interest in its anticancer activity was further investigated. Another cembranolide compound, sinularin, isolated from *S. flexibilis* collected in Hayman Island on the Great Barrier Reef of Australia has shown confirmed antineoplastic activity in the National Cancer Institute’s in vitro bioassay against P-388 lymphocytic leukemia ED50, 7.0 μg/mL [105]. A total of five cembranolides, namely 11-epi-sinulariolide acetate **15**, 11-dehydrosinulariolide **16**, sinulariolide **14**, dihydrosinularin **17**, and 3,4:8,11-bisepoxy-7-acetoxycembra-15(17)-en-1,12-olide **18**, were isolated from *S. flexibilis* (Figure 1) [106]. It has been determined that the bioactivities are diminished or reduced for compounds containing a hydrogenated α-methylene-lactone system, an epoxidated double bond at positions C-7 and C-8, and/or an ether linkage between C-8 and C-11. The α-methylene-lactone system has been found to have a significant impact on cytotoxicity [106].

**Figure 3:**
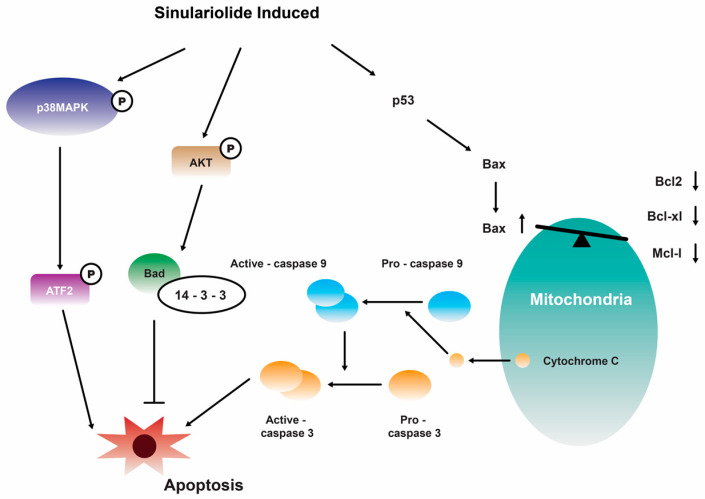
Illustration of how sinulariolide **14** induces cellular apoptosis through mitochondrial and p38MAPK-related pathways in TSGH cells [107]. Reproduced from Neoh, C.A.; Wang, R.Y.; Din, Z.H.; Su, J.H.; Chen, Y.K.; Tsai, F.J.; Weng, S.H.; and Wu, Y.J. “Induction of apoptosis by sinulariolide from soft coral through mitochondrial-related and p38MAPK pathways on human bladder carcinoma cells”, published by MDPI, *Marine Drugs*, 2012.

Further investigation showed that the growth and migration of bladder carcinoma cells were effectively suppressed by **14** in a dose-dependent manner. This inhibition was found to be associated with apoptosis, specifically through the activation of caspase-dependent pathways that are mediated by mitochondria. The observed apoptotic process involved various molecular events, including the loss of mitochondrial membrane potential, release of cytochrome C, activation of caspase-3/-9, Bax, and Bad, as well as the suppression of Bcl-2/Bcl-xL/Mcl-1. Furthermore, the suppression of p38MAPK activity results in the restoration of cellular viability in sinulariolide-treated TSGH cells, suggesting that the p38MAPK pathway is also implicated in the sinulariolide-induced cellular apoptosis (Figure 3) [107]. Collectively, the findings of this study indicate that **14** elicits apoptosis in bladder cancer cells via mechanisms associated with mitochondria and the p38MAPK signaling pathway [107]. Consistent with the findings of previous studies, it was observed that the inhibition of TSGH-8301 cell migration or invasion exhibited a concentration-dependent relationship, suggesting that the treatment with **14** resulted in the downregulation of phosphorylated proteins associated with the mTOR signaling pathway [108]. Therefore, it can be concluded that **14** exhibits significant antitumorigenic properties when tested on cells of bladder cancer. 

**Figure 4:**
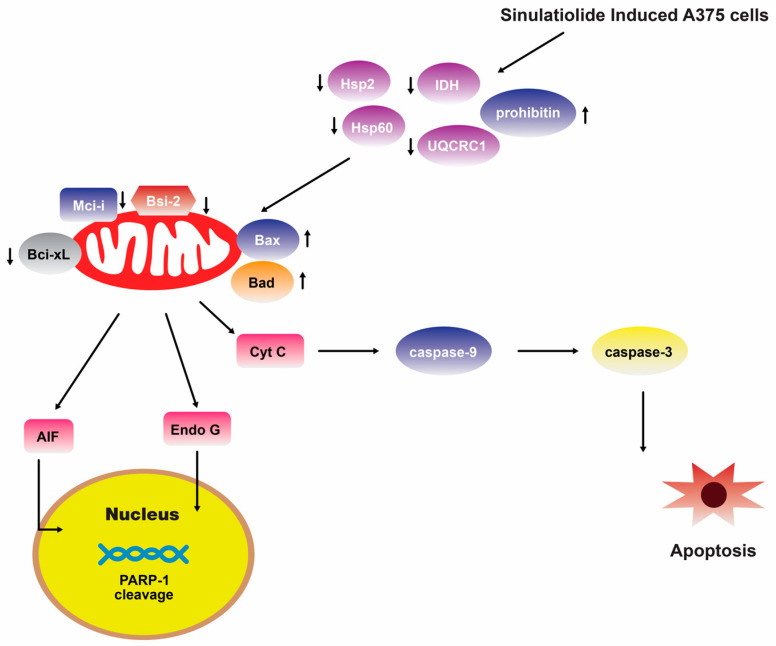
Sinulariolide **14** induces A375 melanoma cell apoptosis through the mitochondrial-related apoptosis pathway and activation of the caspase cascade [109]. Reproduced from Li, H.H.; Su, J.H.; Chiu, C.C.; Lin, J.J.; Yang, Z.Y.; Hwang, W.I.; Chen, Y.K.; Lo, Y.H.; and Wu, Y.J. “Proteomic investigation of the sinulariolide-treated melanoma cells A375: effects on the cell apoptosis through mitochondrial-related pathway and activation of caspase cascade”, published by MDPI, *Marine Drugs*, 2013.

**Figure 5:**
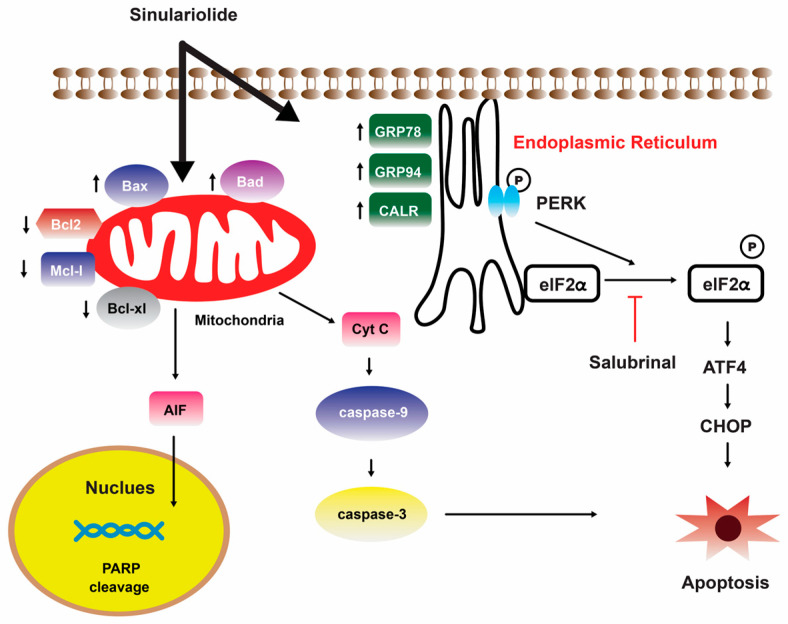
Illustration of how sinulariolide **14** induces cellular apoptosis through mitochondrial-related apoptosis and the PERK/eIF2α/ATF4/CHOP pathway on HA22T cells [110]. Reproduced from Chen, Y.J.; Su, J.H.; Tsao, C.Y.; Hung, C.T.; Chao, H.H.; Lin, J.J.; Liao, M.H.; Yang, Z.Y.; Huang, H.H.; Tsai, F.J. et al. “Sinulariolide induced hepatocellular carcinoma apoptosis through activation of mitochondrial-related apoptotic and PERK/eIF2α/ATF4/CHOP pathway”, published by MDPI, *Molecules*, 2013.

A separate scientific investigation was conducted to examine the impact of **14** on melanoma A375 cancer cells. The findings of this study suggest that **14** promotes apoptosis in A375 cells through the intrinsic mitochondria dysfunction pathway and the activation of the caspase cascade (Figure 4) [109]. In contrast, various cytotoxic mechanisms of **14** towards hepatocellular carcinoma cells have been postulated. It exhibited a dose-dependent inhibition of the growth and ability to form colonies in HCC HA22T hepatocellular carcinoma cells. Additionally, it induced both early and late stages of apoptosis [110]. Figure 5 depicts the pathways involved in mitochondrial-related apoptosis and the activation of the PERK/eIF2α/ATF4/CHOP signaling cascade triggered by **14** [110]. Furthermore, the findings from the zymography assay demonstrated that **14** exhibited inhibitory effects on the enzymatic activities of matrix metalloproteinase (MMP)-2 and MMP-9. Moreover, protein levels of MMP-2, MMP-9, and urokinase-type plasminogen activator (uPA) were reduced by **14** in a concentration-dependent manner [111]. The obtained results indicate that the cytotoxicity of **14** on hepatoma cells is mediated through the activation of multiple apoptotic signaling pathways.

## 4. Tunicates

### Trabectedin

Sixteen years ago, the European Union granted approval for the use of trabectedin **19** (Figure 1) (commercially known as Yondelis and developed by PharmaMar), marking a significant milestone as the first marine-derived anticancer medication authorized for the treatment of soft-tissue sarcoma. This specimen was obtained from the tropical sea squirt and Caribbean tunicate species *Ecteinascidia turbinate* [112]. The chemical structure of **19** is distinguished by the presence of three fused tetrahydroisoquinoline subunits. Compound **19** forms covalent bonds with guanine residues located in the minor groove of the DNA double helix. This interaction initiates a series of sequential processes that involve interference with various transcription factors, DNA-binding proteins, and repair mechanisms, ultimately leading to the disruption of the cell cycle [113]. In addition, it is important to showcase the mechanism by which Yondelis^®^, in addition to triggering the caspase-8-dependent cascade of apoptosis, enhances the susceptibility of cancer cells to Fas-mediated cell death at concentrations that can be feasibly attained and are comparable to those observed in the plasma of patients [114,115].

A study was conducted in 2009 to investigate the in vitro metabolism of **19** (Yondelis^®^) in both monkeys and humans [115]. Most metabolic transformations took place within the trabectedin A domain. These metabolic transformations encompassed mono-oxidation and di-oxidation, as well as the formation of carboxylic acids with or without further oxidation. Additionally, demethylation reactions occurred, leading to the production of ET-729 through N-demethylation, either independently or in conjunction with mono-, di-, or tri-oxidation processes [115]. An additional metabolite was observed because of *O*-demethylation at the trabectedin C subunit. Furthermore, the aliphatic ring opening of the methylene dioxybridge at the B domain was also identified [115]. In the concurrent year, it was documented that **19** has potential antineoplastic properties against soft tissue sarcomas [116]. Individuals diagnosed with myxoid liposarcoma (MLS), a specific subtype distinguished by the presence of the oncogenic transcript FUS-CHOP, exhibit a notable and favorable response to **19** treatments [116]. Patients with MLS are highly responsive to **19** [117].

The feasibility of utilizing **19** in patients with advanced solid tumors was assessed through Phase I clinical trials. The results demonstrated a complete response in patients specifically diagnosed with sarcoma [118]. Studies have demonstrated promising outcomes in the treatment of myxoid liposarcoma through the utilization of Phase I and II clinical trials. These trials have revealed significant response rates, which can be attributed, at least in part, to the suppression of the FUS-CHOP transcription factor [119]. Ongoing Phase II trial investigations are being conducted to explore the phenomenon of locally relapsed uterine leiomyosarcoma [120,121] as well as soft-tissue sarcomas [122], with a focus on clinical development and evaluation.

**Figure 6:**
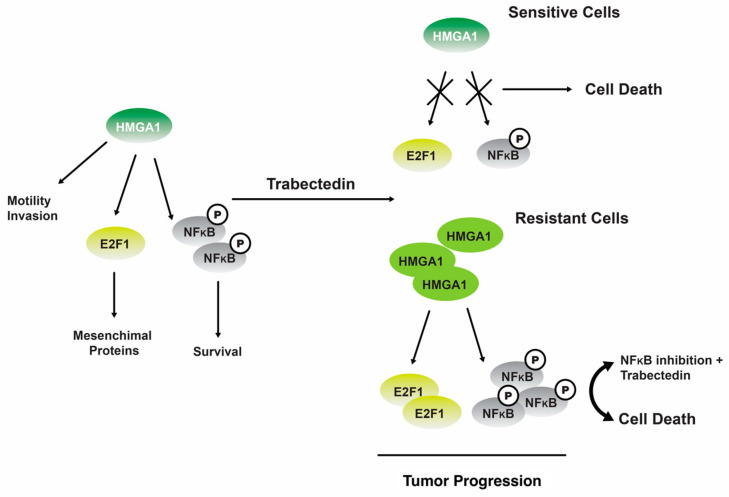
The interplay between the HMGA1 and NFkB signaling pathways trabectedin **19** exerts its inhibitory effects on HMGA1 expression and function by binding to the minor groove of DNA in sensitive cells, ultimately leading to cellular apoptosis. In cells that exhibit resistance, the administration of **19** leads to upregulation of HMGA1 expression and activation of NFkB, thereby promoting the progression of tumors [124]. Reproduced from Loria, R.; Laquintana, V.; Bon, G.; Trisciuoglio, D.; Frapolli, R.; Covello, R.; Amoreo, C.A.; Ferraresi, V.; Zoccali, C.; Novello, M. et al. “HMGA1/E2F1 axis and NFkB pathways regulate LPS progression and trabectedin resistance”, published by Springer Nature, *Oncogene*, 2018.

One study revealed that **19** can regulate NF-kB transcriptional activity in tumor cells that have undergone senescence. The phenomenon leads to perturbation of the equilibrium between antiapoptotic and proapoptotic signaling pathways, thereby rendering cells more susceptible to Fas-induced apoptosis [123]. The administration of **19** has been observed to induce a downregulation of the gene expression for the nonhistone chromatin structural protein, HMGA1, at the transcriptional level in trabectedin-sensitive MLS cells. However, this downregulation effect is not observed in trabectedin-resistant MLS cells (Figure 6) [124].

Consistent with the findings reported in other types of tumors, it was postulated that the occurrence of DNA damage leads to an upregulation of ATM (ataxia telangiectasia mutated) kinase, which subsequently triggers the transcription of HMGA1 [124]. In cells that exhibit resistance, the administration of **19** does not elicit any discernible impact. However, the inhibition of nuclear factor kappa B (NFkB) by inhibitor of kappa B (IKB) partially reinstates their sensitivity to **19** therapies [124].

## 5. Marine Mollusks

### 5.1. Kahalalide F

Mollusca is widely acknowledged as the second most taxonomically diverse phylum in the animal kingdom, encompassing over 85,000 distinct species that have been classified into 8 distinct classes [125,126]. Additionally, it comprises the most extensive assemblage of biologically toxic invertebrates, inhabiting various ecological niches, including marine, freshwater, and terrestrial ecosystems [125,127]. The remarkable capacity for adaptation exhibited by these organisms implies the presence of a highly efficient biosynthetic pathway responsible for the synthesis of a wide range of bioactive compounds. These compounds have demonstrated various beneficial properties, including anti-tumor, antileukemic, antibacterial, and antiviral activities [125,128].

Kahalalide F **20** (Figure 1) is a bioactive compound derived from mollusks that holds considerable significance in the biomedical domain, specifically in the field of cancer therapy. In 1963, Scheuer and colleagues successfully isolated a peptide from the marine mollusk *Elysia rufescens*, a herbivorous species found in Hawaii. This peptide, derived from marine sources, possesses dehydroaminobutiric acid within its molecular structure (Figure 1) [129]. Subsequently, **20** was extracted in limited quantities from the diet of green algae (*Bryopsis* spp.) consumed by the organisms [129]. The synthesis of **20** in response to the diet can be attributed to the mollusk’s ability to acquire and retain chloroplasts from its algal diet. This process is followed by the activation of biosynthetic pathways responsible for producing secondary metabolites [130]. Compound **20** was observed to impede the progression of the cell cycle from G0 to G1 phase in diverse tumor cell lines, including prostate (DU145), cervical (HeLa), colon (HT29), and head and neck (HN30) cell lines [131]. The ability of **20** to disrupt membranes can be attributed to its hydrophobic nature [132]. Findings on the origin of **20** suggested that this potent cytotoxic compound comes from a bacterial origin. Through metagenomic analysis, it was found that the obligate bacterial symbiont *Candidatus endobryopsis kahalalidefaciens* of the green algae produces this compound, which protects the host from predators and is thereafter utilized by the predator (*E. rufescens*) for its own defense [133,134].

The in vitro cytotoxicity effect of **20** was found to be not schedule-dependent against tumor specimens from human patients, namely breast, colon, non-small cell lung, and ovarian tumors, at a concentration of 1 µM [135]. In addition, the cytotoxicity of **20** was observed in vitro against mesenchymal chrondrosarcoma and osteosarcoma cells [136]. Furthermore, it has been observed that tumor cells exhibiting elevated levels of HER-2/neu and/or HER3 expressions display a heightened sensitivity to **20**. This sensitivity is attributed to caspase-independent cell death, as well as the activity of cathepsin B or D, independent apoptosis, and the downregulation of AKT signaling [137]. Hormone-independent prostate tumors, as well as neu+ (HER2-overexpressing) breast cancer tumors and neuroblastoma in animal models, have demonstrated sensitivity to **20**, exhibiting an IC50 value of less than 1 µM. Moreover, these findings indicate that the maximum tolerated dose in animal models is 300 µg/kg (equivalent to 1800 µg/m^2^). Nevertheless, administration of a single dose of 300 µg/kg resulted in organ toxicity in Phase I trial, specifically impaired renal function attributed to damage in the distal convoluted tubules. Moreover, the presence of necrotizing inflammation in the bone marrow and an increase in pretrabecular osteocyte proliferation were also documented [138].

Preliminary investigations in Phase I clinical trials involving patients with androgen-refractory prostate cancer have indicated that a dosage of 560 µg/m^2^/day is the highest dose that can be tolerated. Furthermore, based on the studies conducted on malignant melanoma, metastatic solid tumors, and metastatic lung adenocarcinoma, it has been proposed that their maximum tolerated doses are 800 µg/m^2^ [137,138], 1200 µg/m^2^, and 6650 µg/m^2^ [139], respectively. The administration of once-a-week therapy involves infusing doses for a duration of 1 h. However, it is important to note that there were instances of dose-limiting toxicities observed. These toxicities manifested as asymptomatic and reversible grade 3 or 4 elevations in transaminase blood levels. The observed adverse events included fatigue, paresthesia, pruritus, and nausea, among others. However, it is worth noting that prolonged disease stabilization for a duration exceeding one month was also observed [138,139]. The Phase II clinical trial findings suggest that a dose of 650 µg/m^2^ administered through a 1 h infusion is recommended for once-a-week therapy [138]. PharmaMar, the entity responsible for the development of **20**, entered into a global licensing agreement with Medimetriks in 2009. This agreement pertained to the utilization of **20** and three of its analogs, specifically excluding their application in the fields of oncology and neurology research. Furthermore, the synthetic derivative of **20** that is currently available in the commercial market is referred to as elisidepsin (PM02734, Irvalec^®^) [140].

### 5.2. Spisulosine

Spisuline is a compound that has a structure similar to the phospholipid sphingosine. It was found in the sea mollusk *Spisula polynyma* and has been shown to kill PC-3 and LNCaP cells from the prostate [141]. Interestingly, spisulosine and its diastereo- and regio-isomers have been successfully synthesized via a series of high-yielding chemical steps and high enantioselectivity methods, starting with palmityl alcohol [142]. A team of researchers from PharmaMar conducted preliminary studies on the antiproliferative property of compound ES-285, or also known as spisulosine **21** (Figure 1), isolated from *S. polynyma* [143]. The compound **21** was granted a license by PharmaMar and has been widely employed in clinical research. Compound **21** exhibited potential anti-tumor properties by reducing cell focal adhesion. This cellular process plays a crucial role in the progression of cancer. It has been hypothesized that the molecular target of **21** may be the GTP-binding protein involved in the regulation of actin stress fibers [144]. Furthermore, it has been discovered that **21** exhibits the ability to induce the activation of caspases 3 and 12 signaling pathways, along with the poly ADP-ribose polymerase pathway and p53 phosphorylation [145]. Additionally, it has been discovered that **21** functions as a suppressor of proliferation in prostate tumor cells. Importantly, this inhibitory effect is not mediated by stress-induced mitogen-activated protein kinases (MAP kinases) or peroxisome proliferator-activated receptor gamma (PPARγ) receptors. Furthermore, spisolusine’s mechanism of action is not dependent on the phosphoinositide 3-kinase (PI3K)/protein kinase B (Akt) pathway or classical protein kinase Cs (PKCs). Notably, **21** also stimulates the production of ceramide, a lipid molecule involved in various cellular processes [141]. In addition, **21** exhibited selectivity as a CK1e inhibitor and demonstrated antiproliferative activity against various cancer cell lines including human breast cancer cells (HBL-100 and T-47D), human cervical carcinoma (HeLa), alveolar cell carcinoma (SW1573), and colorectal adenocarcinoma (WiDr) [146]. Compound **21** was observed to induce significant changes in the physical structure of Vero cells, with an IC50 value of 1 µM [143]. Furthermore, **21** demonstrated a greater inhibitory impact on androgen-resistant PC-3 cells compared to androgen-responsive LNCap cells [141]. Additionally, **21** exhibited cytotoxic effects against P-388 (0.01 µg/mL), HT-29 (0.05 µg/mL), and MEL-28 (0.05 µg/mL) tumor cell lines in in vitro experiments [147]. Notably, **21** was additionally observed to demonstrate encouraging selective toxicity against MCF-7, CaCo-2, HCT116, Jurkat, and HeLa cell lines [148]. Considering numerous preclinical investigations indicating its potential as an antitumor agent, **21** underwent Phase I clinical trials involving patients afflicted with established solid tumors. These trials employed various dosing regimens, including a 24 h intravenous infusion administered every three weeks [149], as well as a 3 h intravenous infusion administered for five consecutive days every three weeks [143]. Nevertheless, notwithstanding the infrequent occurrences of drug-induced grade 3 or 4 adverse effects and a highly encouraging pharmacokinetic profile, the potential of **21** as an anticancer agent was impeded by dose-dependent hepatic and neurotoxicity; hence, its further development was terminated [144,149]. In terms of its chemical composition, **21** possesses a remarkably uncomplicated molecular structure. This characteristic has rendered it a valuable model for the exploration of novel synthetic methodologies in the quest for spisulosine analogues that exhibit promising bioactivity [150]. Multiple methodologies were explored in the investigation, including techniques utilizing chiral precursors derived from sugars and amino acids [146], non-chiral precursors [143], organocatalysis [151], and synthesis that employed alpha-hydroxylation of aldehydes via proline-catalyzed amnoxylation utilizing nitrosobenzene, subsequently followed by a reduction of the N-O bond [152]. According to previous studies, **21** is one of the significant compounds that has drawn a lot of attention among the deoxyshingosoid-based natural product compounds that have acquired attention over the recent few decades. The compound is currently undergoing Phase 1 clinical trials; however, the human trials were discontinued due to its poor clinical outcomes, which included neurological diseases such as headache, dizziness, neuropathic pain, and diminished consciousness, as well as drug-related central neurotoxicity that resulted in the death of one patient [14]. This opens the door for medicinal chemists to investigate (2S,3S)-2-amino-1-(4-methoxyphenyl)octan-3-ol, a derivative of **21**. The synthetic analog of **21** showed promising inhibitory properties against multiple cancer cell types, including DLD-1, HT-29 (colorectal adenocarcinoma), MCF-7, MDA-MB231 (breast adenocarcinoma), A549, and NCI-H358 (lung carcinoma). When it comes to colon cancer cells (Colo-205, LOVO, HT-29, DLD-1, SW-48, and SW-620), the (2S,3S)-2-amino-1-(4-methoxyphenyl)octan-3-ol has an IC50 value of less than 5 µM. The compound **21** derivative, however, did not show cytotoxicity by traditional apoptosis. This result was in contrary to its parent chemical **21**, which showed an active apoptotic impact by increasing Annexin-V positive cells as well as PARP cleavage. Instead, (2S,3S)-2-amino-1-(4-methoxyphenyl)octan-3-ol induces autophagic cell death [14]. Homosapisulosine [(3S,4R)-3-aminononadecan-4-ol] was another recently synthesized spisulosine analog that was made from 3,5-di-*O*-benzyl-d-xylofuranose using a stereoconvergent method. By fragmenting and losing cellular DNA, homospisulosine causes cervical cancer cells (HeLa cells) to undergo apoptosis. In addition, it causes the cleavage of its target PARP protein, activates caspase-3, and externalizes phosphatidylserine. However, homospisulosine may dissipate the mitochondrial membrane, which ultimately results in the formation of mitochondrial permeability transition pores, in contrast to the parent molecule **21**, which induces apoptosis by an endoplasmic reticulum stress-mediated process without compromising mitochondrial integrity [153].

### 5.3. Jorumycin

Jorumycin **22** (Figure 1), an additional anti-tumor compound derived from mollusks that exhibits notable antibacterial properties as well. Compound **22** was initially extracted in 1990 from the mantle and mucus of the Pacific nudibranch species, *Jorunna funebris* [154]. This compound is a member of the tetrahydroisoquinoline alkaloid family, which exhibits structural similarities to saframycins, renieramycins, and ecteinascidin [155]. Compound **22** exhibits significant efficacy against NIH 3T3 fibroblast cells, completely inhibiting their growth at a concentration of 50 ng/mL. Furthermore, it demonstrates notable activity against P388, A549, HT29, and SK-MEL-28 lung tumor cell lines, with an IC50 value of 12.5 ng/mL [154]. Compound **22** can be synthesized through a synthetic process that involves utilizing L-tyrosine as the chiral starting material, along with its analogues. These analogues have been discovered to exhibit in vitro cytotoxic activity against various cell lines, including HCT-8 (colon), BEL-7402 (liver), Ketr3 (kidney), A2780 (ovary), MCF7 (breast), A549 (lung), BGC-823 (stomach), HeLa (cervix), human embryonic lung fibroblast (HELF), and human oral epidermoid carcinoma KB cells [156]. Furthermore, it demonstrates significant suppression of tumor growth in human colon (HCT116) and lung (A549) cell lines, with a GI50 ranging from 1.9 to 24.3 nM. However, its 3-*epi*-jorumycin analogue exhibits lower cytotoxicity, with a GI50 ranging from 0.6 to 14.0 µM [157]. Furthermore, the hippuric acid derivative analogues demonstrated significant and wide-ranging cytotoxic effects against ten different cell lines (HCT-8, BEL-7402, Ketr3, A2780, MCF-7, A549, BGC-823, HeLa, HELF, and KB). The average IC50 value for these analogues was determined to be 2.12 nM [155]. Because of its potential as an anti-tumor agent, several syntheses were completed, where Zalypsis^®^ **23** (Figure 1) was the most important and well-known compound [158]. Zalypsis^®^ **23** is a synthetic dimeric tetrahydroisoquinoline that is currently undergoing clinical trials for its potential therapeutic efficacy in the treatment of solid tumors and hematologic malignancies [158,159,160]. Compound **23** exerts its apoptotic activity by inhibiting the cell cycle and transcription processes, specifically by inducing breakage of the DNA double strand. This DNA damage subsequently leads to the accumulation of cells in the S phase of the cell cycle [159,160]. The average half-maximal inhibitory concentration (IC50) of **23** across a set of 24 cell line panels is determined to be 7 nM. Notably, among these panels, the leukemia and stomach tumor cell lines exhibited the highest sensitivity to **23** [159]. Additionally, **23** exhibits noteworthy suppression of tumor growth in murine xenograft models of human cancer, as well as in xenograft tumors of human transplanted breast, gastric, prostate, and renal origin [161]. Compound **23** has demonstrated efficacy against multiple myeloma cell lines MM.1R and RPMI-8226/LR5, both of which are known to be resistant tumor cell lines. This finding implies that **23** may have potential as a therapeutic agent for the treatment of drug-resistant tumors [158]. Compound **23** underwent Phase I clinical trials to evaluate its efficacy in treating solid tumors and lymphoma [158,161]. According to the findings from Phase II clinical trials, it has been observed that **23** exhibits significant antimyeloma properties. Its potency is particularly noteworthy, as evidenced by its IC50 values ranging from picomolar to nanomolar. Mechanistically, this agent exerts its effects by impeding the cell cycle and triggering apoptotic cascades. These cascades are characterized by a decrease in the cellular population in the G2/M phase, an increase in the G0/G1 phase, and the downregulation of various genes associated with cell cycle progression [158]. The most frequently observed negative effects of this treatment consist of fatigue, loss of appetite, nausea, an increase in troponin I levels, and neutropenia. These effects were temporary and could be effectively managed through adjustments in dosage or delays in administration [162]. The recommended daily dosage for this treatment is 2 mg/m^2^ [163,164,165]. Nevertheless, the efficacy of **23** in treating Ewing sarcoma [164], urothelial carcinoma [164], endometrial cancer, and cervical cancer [165] remains ambiguous based on the findings of its Phase II clinical trials. In addition, three recent clinical trials were conducted for **23**. Two of these studies were discontinued because of inadequate and poor patient recruitment. The results of **23**’s clinical trial, which began in October 2021, were published for patients with advanced and/or metastatic endometrial or cervical cancer who had previously had one line of systemic chemotherapy (NCT01222767). Nevertheless, this study’s findings have not been released yet [166].

## 6. Ascidians

### 6.1. Meridine

**Figure 7:**
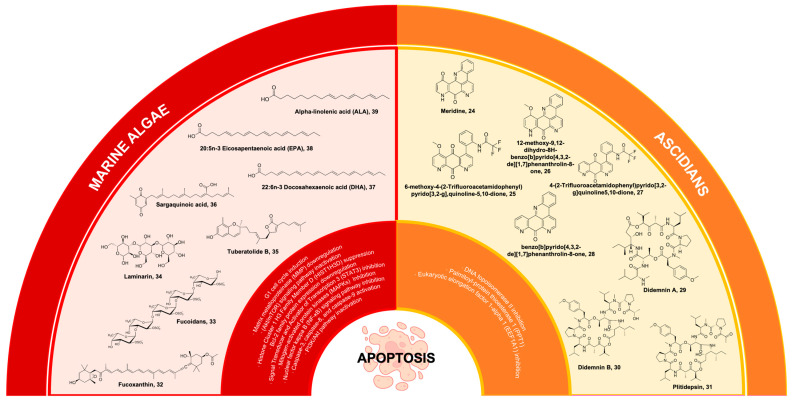
Marine natural products from algae and ascidians and their synthetic derivatives, with their mechanisms of action targeting the apoptosis pathways.

Ascidians represent a prolific reservoir of pharmacologically potent secondary metabolites, which exhibit considerable potential as drug candidates for the therapeutic intervention of diverse health ailments, with a particular emphasis on cancer [167,168]. One of the bioactive compounds derived from ascidians is meridine **24** (Figure 7), which has been extracted from *Amphicarpa meridian*. Compound **24** has demonstrated anticancer properties by effectively inhibiting topoisomerase II activity. This inhibitory effect is observed at a relatively low concentration range, spanning from 10 mM to 10 nM [169,170]. Moreover, it was also reported that **24** exhibits cytotoxicity against metastatic human bladder cancer cell lines (TSU-Pr1, TSU-Pr1-B1, and TSU-Pr1-B2) and the superficial bladder cancer cell line 5637 with an IC50 ranging from 3.76 to 4.56 µM against the TSU-Pr1 series and 10 µM against 5637 cell lines [171]. As a result, analogues were derived from the meridine parent molecule to enhance the compound’s anticancer activity. A total of twenty-four (24) analogues were acquired through substitutions on ring A, primarily focusing on the R1 position. All the analogues demonstrate cytotoxicity, with certain analogues exhibiting cytotoxicity levels that are 10,000 times greater than **24** when tested against a panel of 12 human cancer cell lines [169]. These compounds were 6-methoxy-4-(2-trifluoroacetamidophenyl)pyrido[3,2g], quinoline-5,10-dione **25**, **26** (differing from **25** as it exists as a pentacyclic derivative), 4-(2-Trifluoroacetamidophenyl)pyrido[3,2-g]quinoline-5,10-dione **27**, and benzo[b]pyrido[4,3,2-de][1,7]phenanthrolin-8-one **28** (Figure 7) [169,170]. Additionally, **24** exhibits characteristics of a DNA intercalator, displaying robust quadruplex ligand activity as assessed using a TRAP (telomerase repeated amplification protocol) assay. This finding further supports its potent inhibitory effect on telomerase activity [172].

### 6.2. Didemnins

Didemnins are ascidian-derived compounds that are currently being studied for their potential in cancer treatment. These compounds have been extracted from the Caribbean tunicate *Trididemnum solidum* [173]. They exhibit significant activities against tumors, viruses, and immune system suppression [174]. Among the compounds belonging to the didemnin class, didemnin A **29** (Figure 7) possesses the most straightforward molecular structure and is widely prevalent. However, **29** exhibits the lowest level of biological activity. Likewise, it should be noted that among the group didemnin B **30** (Figure 7) exhibits the highest abundance and activity levels [175]. Compound **30** is a cyclic peptolide that has been branched and N-methylated. It has been observed to possess remarkable anticancer properties in animal models [176]. Compound **30** demonstrates a swift induction of apoptosis through the inhibition of palmitoyl-protein thioesterase 1 (PPT1) and eukaryotic elongation factor 1-alpha 1 (EEF1A1) [177].

Compound **30** holds the distinction of being the first marine-derived compound to undergo clinical trials in its pure form [173,174,178]. Nevertheless, the unfavorable outcomes observed in Phase II clinical trials conducted on various cancer cell lines, such as Hodgkin’s lymphoma, coupled with the severe and potentially lethal side effects associated with **30**, have significantly deterred further exploration into its potential [175]. Because of this factor, dehydrodidemnin B (aplidine), a compound analogous to **30**, has emerged as the most auspicious candidate among the didemnins. Notably, dehydrodidemnin B is derived from the polyclinidae family, in contrast to the didemnidae family [174]. Plitidepsin **31** (Figure 7), with the trade name Aplidin^®^, is a cyclic depsipeptide that has been extracted from the Mediterranean tunicate *Aplidium albicans* [179]. At lower concentrations, **31** demonstrates notable antineoplastic effects against breast cancer, melanoma, and non-small cell lung cancer. The mechanism of action of this phenomenon encompasses multiple pathways, including cell cycle arrest and the inhibition of protein synthesis, among other factors [176]. The findings from Phase II clinical trials indicate that combining **31** with dexamethasone, as opposed to using dexamethasone alone, shows promising outcomes as a treatment for patients with relapsed or refractory multiple myeloma [179]. However, it is worth noting that a temporary increase in transaminase levels, leading to muscle and liver toxicities, was observed as the main limitation in terms of dosage for **31** [180,181]. Compound **31** exhibited anti-tumor effects in xenograft multiple myeloma models, and a combination of **31** and dexamethasone showed activity in relapsed/refractory multiple myeloma in Phase II clinical trials. Moreover, in the Phase III ADMYRE trial of combination therapy, Australia approved the combination treatment for patients with relapsed/refractory multiple myeloma who received prior regimens and for patients who are intolerant to both protease inhibitors and immunomodulatory drugs [182,183,184]. In a recent Phase I trial conducted in Spain and France, 36 patients with relapsed/refractory multiple myeloma (r/r MM) were treated with **31**, bortezomib (BTZ), and dexamethasone (DXM). Using a typical dosage escalation methodology, the highest dose level assessed (aplidin 5.0 mg/m^2^, BTZ 1.3 mg/m^2^, and DXM 40.0 mg) was identified as the optimal dose for Phase II research. In addition to tolerable hematological toxicities, two patients suffered dose-limiting toxicities (grade 3 diarrhea and grade 3 nausea/vomiting). The overall response rate was 22.2% in the suggested dose cohort, while the clinical benefit rate was 77.8%. In adult patients with r/r MM, the triple combination showed a modest level of activity and an acceptable safety profile [185].

## 7. Marine Algae

### 7.1. Fucoxanthin

Carotenoids represent a collection of naturally occurring pigments encompassing both nonpolar hydrocarbon carotenes and polar compounds known as xanthophylls. These entities possess numerous biological functions, including the scavenging of free radicals, regulation of the immune system, scavenging of singlet oxygen, and various pharmacological effects [186]. Fucoxanthin **32** is a secondary carotenoid found in brown algae that constitutes more than 10% of the overall carotenoid content found in nature, particularly in marine ecosystems [187]. Compound **32** is a xanthophyll compound, characterized by its orange coloration. Its unique structure is notable for containing an uncommon allenic bond, an epoxide group, and a conjugated carbonyl group within a polyene chain (Figure 7) [188].

In addition to its well-documented antioxidant properties [189], **32** has demonstrated significant apoptotic effects on various carcinoma cell lines. These include prostate cancer cell lines (PC-3, DU145, LNCaP), leukemia cell lines (HL-60, HP50-2, HP100-1, ATL), colon cancer cell lines (HT-29, caco-2, DLT-1, LS1174T), liver cancer cell lines (HepG2, S-Hep-1), urinary bladder cancer cell line (EJ-1), gastric cancer cell line (MGC-803), breast cancer cell line (MCF-7), melanoma cell line (B16F10), and lymphoma cell line (PEL). The anticancer effects of **32** were attributed to disrupted molecular mechanisms of tumor growth and development [190]. Compound **32** demonstrated antitumoral actions, including G1 cell cycle arrest, apoptotic induction, and DNA damage [191]. The impact of **32** on normal cells was either negligible or less pronounced compared to cancer cells, suggesting a distinct and preferential influence of **32** on cancer cells [192]. Compound **32** reduced the viability of U251 and U87 glioma cancer cells as mediated by the compound’s pro-apoptotic, antimigration, and anti-invasion properties. Importantly, these activities were observed without any detrimental effects on normal cells, thus highlighting the potential therapeutic value of **32** in the context of glioma treatment [193]. Recent investigations have further demonstrated compound **32** as a potential anticancer lead against multiple carcinomas. Compound **32** facilitated apoptosis and downregulated MMP gene expression in tongue cancer cell lines (CAL-27) [194]. Additionally, it demonstrated antiproliferative properties against CAL-27 cell lines through alteration of glucose uptake and inactivation of protein kinase B/mammalian target of the rapamycin (Akt/mTOR) signaling pathway [195]. Moreover, compound **32** could promote cervical cancer cell (HeLa and SiHa) proliferation by suppressing histone Cluster 1 H3 Family Member D (HIST1H3D) expression and arresting the G0/G1 cell cycle [195].

The in vivo efficacy of **32** in inhibiting cancer growth was further substantiated through observations of the reduced weight and volume of glioma tumors in mice. The metabolic pathway of **32** in mice and HepG2 cells was elucidated, demonstrating its enzymatic conversion into two distinct metabolites, namely fucoxanthinol and amarouciaxanthin A [193]. Compound **32** undergoes hydrolysis in the gastrointestinal tract prior to its absorption in the intestine. This hydrolysis process results in the formation of fucoxanthinol. Subsequently, **32** is metabolized in the liver, where it is converted into amarouciaxanthin A [196].

Research efforts have been initiated to identify anticancer compounds that possess standalone efficacy or synergistic potential when combined with other chemotherapeutic agents. These strategies aim to enhance the therapeutic outcomes of cancer treatment while simultaneously mitigating its adverse effects. The antimetabolite known as 5-fluorouracil (5-Fu) has been widely utilized as a chemotherapeutic agent for the treatment of colorectal cancer [197]. Nevertheless, the upregulation of the enzyme thymidylate synthase and modifications in signaling cascades governing cellular proliferation and programmed cell death play a significant role in the emergence of resistance towards 5-Fluorouracil (5-Fu). The investigation of **32**’s anticancer properties, both independently and in conjunction with 5-Fu, has emerged as a potentially effective therapeutic approach considering the increasing resistance of colon cancer cells to 5-Fu [198]. The administration of **32** as a standalone treatment resulted in a reduction in the viability of cells. Furthermore, when **32** was combined with 5-Fu, it exhibited an augmented cytotoxic impact on HCT116 and HT29 colon cancer cell lines. Importantly, this combined treatment did not exhibit any detectable toxicity in normal cells [198].

In addition, the cytotoxic effects of **32** was investigated both individually and in conjunction with the established antileukemia medications imatinib (Imat) and doxorubicin (Dox) on erythroleukemia (K562) and lymphoblast (TK6) cell lines, which are associated with chronic myelogenous leukemia [199]. This investigation was motivated by the observed adverse effects of Imat and Dox, such as cytotoxicity in healthy cells and the development of resistance to multiple drugs [199]. Compound **32** has the potential to function as a chemotherapeutic adjuvant in the treatment of extremely metastatic triple-negative breast cancer (TNBC) when paired with Dox [200]. On TNBC cell lines (SKBR3 and MDA-MB-231), the combined action of Dox and **32** demonstrated greater cytotoxic activity than tests conducted separately [200]. Compound **32** increased cancer cell mortality, decreased cell proliferation, and caused ultrastructural damage in MDA-MB-231 breast cancer cells, all of which improved the cytotoxic effect of Dox [200]. Moreover, the selective anticancer mechanism of **32** may be used as a supplemental therapy to boost the antiproliferative activity of gemcitabine, a DNA synthesis inhibitor, for pancreatic cancer cell lines (MIA PaCa-2), without endangering non-cancerous cells [201]. These discoveries present a hopeful alternative wherein the utilization of **32** in conjunction with the existing cancer medications demonstrates efficacy against multidrug-resistant cancer cell lines while maintaining the integrity of normal cells.

### 7.2. Fucoidans

Sulfated polysaccharides are essential anticancer macromolecules with a wide range of industrial, biological, and medicinal uses [202,203]. The sulfated polysaccharide fucoidan **33** (Figure 7) is found in the cell surface of brown seaweeds, comprising 5 to 20% of the algae’s dry weight [204]. Fucose and sulfate are the main structural components of the water-soluble polymer **33**, whose structural moieties and branching chains vary depending on the species of algae that was recovered [203,205]. Compound 33 is a key marine algal polysaccharide (MAP) that exhibits remarkable anticancer mechanisms to supplement combination chemotherapy [206].

Due to its therapeutic potential against malignant cells, specifically through cell cycle arrest, apoptosis induction, suppression of angiogenesis, and modulation of inflammatory responses, **33** has been extensively researched for its anticancer efficacy [207]. Compound **33** has demonstrated anticancer activity both in vivo and in vitro against a variety of human cancer cell lines [208], including those from the lung (A549, HMEC-1, and H1650) [209,210,211], the ovary (ES-2 and OV-90) [212], the anaplastic thyroid (FTC133) [213], the breast (MDA-MB-231 and HPMEC-ST1.6R) [214], the colon (HT29) [215], the prostate (DU-145) [216], TNBC (MDA-MB-231 and HCC1806) [217], the liver (SMMC-7721, Huh7 and HCCLM3) [218], the bladder (T24) [219], and the pancreas (MiaPaCa-2, Panc-1, AsPC-1 and BxPC-3) [220]. Furthermore, **33** showed no cytotoxic effect on Vero or HaCaT cells while exhibiting dose-dependent anticancer activity against the lung cancer cell line (A549) [221]. In a recent study, very-low-molecular-weight formulations of **33** (vLMW-F) reduced cell proliferation and induced apoptosis of lymphoblastoid cell lines (LCLs) and diffused large B-cell lymphomas (DLBCLs) [222]. Compound **33** and vLMW-F downregulated the expression of programmed death-ligand 1 (PD-L1), which is overexpressed in latency III B-cells of the oncogenic Epstein–Barr virus (EBV) for suppressing an anti-tumor T-cell response [222]. Without harming non-tumor cells, the low-molecular-weight derivatives of **33** may be used as an adjuvant antiPD-L1 medication with immunotherapy. When used in conjunction with olaparib, the sulfated low-molecular-weight derivative of **33** called oligo-fucoidan enhanced the therapeutic activity of olaparib, the poly (ADP-ribose) polymerase (PARP) inhibitor, as a treatment for triple-negative breast cancer (TNBC) [223].

Several clinical trials were conducted to assess **33**’s anticancer activity in cancer patients. Oligo-fucoidan was supplemented for patients with cancer-induced sarcopenia, metastatic colorectal cancer, and non-small cell lung cancer (NSCLC). The auxiliary effects of **33** in cancer patients with locally advanced rectal carcinoma who had received radio-chemotherapy before surgery was evaluated in observational clinical research. Compound **33** was used in conjunction with radiation and chemotherapy for patients with late-stage (III/IV) head and neck squamous cell carcinoma in a double-blind, randomized Phase II research. ClinicalTrials.gov provides the public with access to these clinical trials. All things considered, these studies showed how compound **33** derived from brown algae could progress the therapy of numerous carcinomas.

### 7.3. Laminarin

Laminarin, or laminaran **34** (Figure 7) found in brown seaweeds, is a functional food that has been considered a major part of the traditional diet in Asian countries. This carbohydrate food reserve in brown algae contains β(1-3)-glucan with β(1-6)-glucan linkages. β-glucan has been associated with stimulating immunity and possessing anti-tumor activity [224,225]. Compound **34** was isolated from *Laminaria japonica* and induced apoptosis in human colon cancer LOVO cells through mitochondrial [226] and death receptor [227] pathways, while the apoptotic activity of **34** isolated from *L. digitata* in HT-29 human colon cells was initiated through the intrinsic apoptotic and ErbB pathways [228]. Compound **34** diminished the Bcl-2 family protein expression responsible for the release of pro-apoptotic factors and inhibited cell cycle progression by regulating the ErbB signaling pathway [228].

Interestingly, the sulfated modification of **34** synthesized by the chlorosulfonic acid-pyridine method enhanced the anti-tumor activity of **34** against LOVO cells compared to **34** alone at the same concentration [229]. Compound **34** sulfate has also been shown to inhibit heparanase activity and tumor metastasis in mouse melanoma cells (B16-BL6) and rat mammary adenocarcinoma cells (13762 MAT) [230]. The native and sulfated **34** isolated from the brown algae *Dictyota dichotoma* protected normal epidermal cells, while only sulfated **34** was able to sensitize melanoma cells to X-ray irradiation, resulting in significant inhibition of cell proliferation, colony formation, and migration of cancer cells [231]. The molecular mechanism of this action was related to the inhibition of MMP-2 and MMP-9 proteinase activity as well as the downregulation of kinases’ phosphorylation of the ERK1/2 signaling cascade [231]. Taken together, the combination of the sulfated derivative of **34** from *D. dichotoma* with X-rays may serve as a potential treatment strategy for human melanoma [231].

Meanwhile, **34** isolated from the brown algae *Eisenia bicyclis* restrained the progressive development of a precancerous lesion, gastric dysplasia, in alpha-1,4-*N*-acetylglucosaminyltransferase (A4gnt) KO mice, a unique animal model for gastric cancer [232]. The potential oral administration of brown seaweed-derived **34** on the development of gastric dysplasia was determined in 12-week A4gnt KO mice [233] because of the absence of α1,4-*N*-acetylglucosamine-capped *O*-glycans in the gastric gland mucin and the spontaneous development of gastric cancer through the hyperplasia–dysplasia–adenocarcinoma sequence. The administration of **34** substantially attenuated gastric dysplasia development and counterbalanced the increased induction of cell proliferation and angiogenesis. Furthermore, **34** treatments effectively overcame the A4gnt KO-induced alteration in the gene expression profile of selected cytokines, as revealed with real-time PCR analysis [233].

Aside from its putative gastro-protective activity, **34** also has the capacity to promote immune responses and protect leukemic BALB/c mice against liver injury. Leukemia was generated in BALB/c mice using murine acute myelomonocytic leukemia WEHI-3 cell lines [234]. The binding of **34** from *Plodia interpunctella* (Pi-N-βGRP) to an amino-terminal β-1,3-glucan binding domain (N-βGRP) induced the formation of a multiple Pi-N-βGRP-containing macrocomplex, an initiating activating signal of serine protease cascades that promote immune responses [235]. Compound **34** significantly increased the NK cell cytotoxic effect in leukemic mice and reduced T-cell proliferation at 5 mg/mL after stimulation but did not significantly affect B-cell proliferation. Compound **34** restored glutamate oxaloacetate transaminase (GOT) (2.5 and 5 mg/mL) and glutamate pyruvate transaminase (GPT) (2.5 mg/mL) levels in leukemic mice at different doses. In serum, the levels of GPT and GOT activity were higher than normal levels, which may reflect hepatic cell destruction [236].

In a separate study, **34** from kelps and curcumenol from *Curcuma zedoaria* were used in combination to inhibit the proliferation and metastasis of hepatoma cells and consequently improve the prognosis in mice bearing hepatoma-22 (H-22) [237]. The combination of curcumenol and **34** inhibited the proliferation, migration, and invasion of human hepatoma HepG2 cells, as shown by reduced levels of pSTAT3 and BCL-2, dose-dependently decreased hydrogen sulfide (H2S) synthetase, and downregulated VEGF and its downstream key genes pAkt and pERK1/2 [237]. The findings of the study demonstrated that the combination of curcumenol and **34** could inhibit the proliferation and metastasis of liver cancer cells in vivo and in vitro by inhibiting endogenous H2S production and downregulating the pSTAT3/BCL-2 and VEGF pathways, providing strong evidence for the application of curcumenol and **34** in liver cancer treatments.

On Vero cells, the β-glucan-rich **34** showed a non-cytotoxic mechanism, which was also seen for the sulfated polysaccharide **33** [221]. Compound **34** caused apoptosis in liver cancer cell lines (Bel-7404 and HepG2) proportionate to its concentration, whereas it inhibited the colon cancer cell line (HCT116) at IC50 values ranging from 51.15 to 162.79 µg/mL [221]. Moreover, **34** may increase the maturation of dendritic cells to boost type 1 T helper (Th1) and cytotoxic T lymphocyte (CTL) immunological responses [238]. These results might point to the possibility of using **34** as a cancer immunotherapy adjuvant. To date, no clinical studies have been conducted to further investigate the anticancer properties of **34**.

### 7.4. Tuberatolide B

The protein known as Signal Transducer and Activator of Transcription 3 (STAT3) has emerged as a significant focus for cancer therapy, primarily because of its involvement in the development of tumors and the progression of malignancy [239]. STAT3 plays a crucial role in regulating vital biological processes such as cell differentiation, proliferation, apoptosis, angiogenesis, metastasis, and immune responses [240]. This is achieved through the modulation of gene transcription by STAT3. Tuberatolide B **35** (Figure 7), a diastereomeric meroterpenoid, was primarily obtained from the Korean marine tunicate, *Botryllus tuberatus* [240,241]. Previous studies on **35** have established its role as an antagonist of the human farnesoid X receptor (hFXR) [241]. Interestingly, **35** was also isolated from the Korean marine algae *Sargassum macrocarpum* and found to possess significant antitumor activity against breast, lung, colon, prostate, and cervical cancer cells [242].

Compound **35** exhibited inhibitory effects on the phosphorylation of STAT3, as well as its transcriptional activity and the expression of downstream target genes including Cyclin D1 (CCND1), MMP-9, Survivin, and Interleukin 6 (IL-6) [243]. Compound **35** elicited reactive oxygen species (ROS) generation in breast adenocarcinoma (MDA-MB-231), lung (A549), and colorectal (HCT116) cell lines. Consequently, reactive oxygen species (ROS) amplified the occurrence of DNA damage through the process of double-strand breakage. This led to the formation of phosphorylated histone H2AX (γH2AX) foci and the phosphorylation of DNA damage-associated proteins, including checkpoint kinase 2 (Chk2) and H2AX. The serine/threonine kinase Chk2 plays a significant role in regulating the cellular response to DNA damage, and its phosphorylation is essential for the phosphorylation of histone H2AX [243]. The discoveries regarding apoptosis have provided initial evidence that **35**, derived from marine algae, possesses strong potential as an anticancer agent. It has been observed that **35** induces apoptosis through the generation of reactive oxygen species (ROS), which in turn inhibits the phosphorylation of STAT3 and promotes DNA damage [243].

The biological activities of **35** were investigated, particularly its anti-inflammatory effect against lipopolysaccharides (LPS), has been prompted by its recognized anticancer properties. This study employs an in vivo zebrafish model to assess the extent of **35**’s anti-inflammatory effect against LPS [244]. In zebrafish subjected to LPS stimulation, the administration of **35** resulted in improved survival rates and a notable reduction in the production of nitric oxide (NO), an inflammatory mediator. Additionally, **35** treatments led to a decrease in the mRNA expression of inducible nitric oxide synthase. Compound **35** exhibited inhibitory effects on the production of nitric oxide (NO), expression of inducible nitric oxide synthase (iNOS), and pro-inflammatory cytokines in both lipopolysaccharide (LPS)-stimulated RAW264.7 macrophage cells and the zebrafish model [244]. These effects were achieved through the inhibition of mitogen-activated protein kinases (MAPKs) and the nuclear factor kappa B (NF-κB) signaling pathway. The collective evidence supports the significance of **35** as both an anticancer compound and an anti-inflammatory agent. Currently, there is a dearth of preclinical studies on compound **35**. Nevertheless, this underscores its potential as a valuable functional food for inflammation management and as a promising therapeutic agent for chemotherapy.

### 7.5. Sargaquinoic Acid

*Sargassum* species, belonging to the phylum Phaeophyta, are a type of brown algae that exhibit a broad geographical distribution across temperate and tropical marine environments. The species belonging to the Sargassaceae family have been identified as producers of sargaquinoic acid, SQA **36** (Figure 7), which is a meroterpenoid compound exhibiting anti-inflammatory [245,246], antiadipogenic [247], and antiproliferative properties. Compound **36**, which was obtained from the marine brown alga *Sargassum macrocarpum*, has been discovered to exhibit unique properties in promoting neurite outgrowth in PC12D cells, a cell line derived from rat pheochromocytoma. This activity is dependent on nerve growth factor (NGF) and represents a novel finding [248].

In addition to its established neuroprotective properties, **36** has exhibited unique pro-apoptotic effects both in laboratory settings (in vitro) and in living organisms (in vivo) [249]. Compound **36** was extracted from the species *S. sagamianum* and was subsequently subjected to purification methods, and its potential cytotoxicity was assessed on a cell line of immortalized human keratinocytes (HaCaT cells) using the MTT assay. The correlation between the cytotoxic effect and apoptosis was examined using a terminal deoxynucleotidyl transferase-mediated nick-end labeling (TUNEL) assay [249]. The results demonstrated that **36** induced apoptosis in a concentration-dependent manner. In an in vitro setting, the administration of **36** resulted in the induction of cellular apoptosis and the subsequent activation of caspase-3, caspase-8, caspase-9, and poly (ADP-ribose) polymerase (PARP) in a manner that was dependent on the concentration of the compound [249]. Compound **36** has been found to induce apoptosis in a highly effective manner by activating two distinct apoptotic pathways [249]. The first pathway, known as the intrinsic pathway, is initiated by caspase-9 within the cell. This activation leads to a permeable change in the outer membrane of the mitochondria, resulting in the release of cytochrome c into the cytosol. The second pathway, referred to as the extrinsic pathway, is initiated by caspase-8. This pathway is responsible for apoptosis triggered by death receptors upon binding to their natural ligands [250]. Both biological pathways necessitate the involvement of caspase-3, an enzyme that catalyzes the cleavage of the death substrate, ultimately leading to the process of programmed cell death, also known as apoptosis [1]. The process of apoptosis is synergistically induced through irradiation with **36** when it is co-administered with ultraviolet B (UVB) radiation. The co-administration of UVB radiation and **36** resulted in an enhanced stimulation of caspase activation [249]. Furthermore, the activation of caspase-3 occurred at an earlier time point (2 μg/mL) compared to the administration of a single dose of **36** (5 μg/mL). The previous study aimed to investigate the apoptotic activity of **36** on hairless mice in an in vivo setting, specifically focusing on the effects of UVB irradiation [249]. UVB radiation has been observed to induce epidermal hyperplasia, characterized by an elevated proliferation of skin cells in the outermost layer of the epidermis. Upon simultaneous exposure to UVB radiation and **36**, the complete epidermal layer and certain regions of the dermis exhibited observable staining, thereby validating the synergistic effects of these two factors in an in vivo setting [249]. The results of the study indicate that the combined action of UVB and **36** can induce apoptosis in a synergistic manner. This suggests that this combination therapy could be a viable approach for the treatment of skin conditions characterized by excessive growth of the epidermis, such as epidermal hyperplasia. Notably, the use of a lower dose of UVB in conjunction with **36** demonstrates the potential of these agents as therapeutic interventions for hyper-proliferative diseases like psoriasis.

The compound **36** was also found in the ethanolic extract derived from *S. serratifolium*. It exhibits notable hypopigmenting effects on B16F10 mouse melanoma cells [251]. Additional research conducted on the **36** has provided insights into its mechanism of action in inhibiting melanin synthesis. It has been observed that **36** exerts its hypopigmenting effects by modulating the activity of various signaling pathways, specifically the cAMP/responsive element binding protein (CREB) and extracellular signal-regulated kinase (ERK)1/2 pathways. These pathways, in turn, lead to the downregulation of microphthalmia-associated transcription factor (MITF) expression in α-melanocyte-stimulating hormone-stimulated B16F10 cells. This discovery highlights the potential of **36** as a promising therapeutic agent for addressing skin hyperpigmentation disorders [252].

In addition to the promising apoptotic impact of **36** on the HaCaT human keratinocyte cell line, encompassing both the extrinsic and intrinsic apoptotic pathways, a thorough investigation was conducted on the cytotoxic mechanism of **36** derived from *S. heterophyllum*, in MDA-MB-231 breast cancer cells [253]. This study demonstrated that the initiation of apoptosis in MDA-MB-231 cells is mediated by the activation of caspase-3, -6, -8, -9, and -13 enzymes. This process is accompanied by the reduction of Bcl-2, a protein that regulates apoptosis in mitochondria [253]. These findings suggest that the cytotoxic effects of **36** involve both the extrinsic and intrinsic pathways of apoptosis. Furthermore, the examination of the cell cycle through flow cytometry, employing propidium iodide staining, demonstrated a modified cell cycle distribution in the cells subjected to a treatment with **36**. An alteration in the Sub-G0 apoptotic population was observed, which substantiates the results obtained through Hoescht/propidium iodide and annexin V staining, as well as the studies on poly(ADP-ribose) polymerase (PARP) cleavage. An observed augmentation in the G1 phase from 49% to 64% was noted at an IC50 of 67 μM, while the apoptotic impact diminishes with escalating **36** concentration. Furthermore, the administration of **36** resulted in a reduction in the G2/M phase of the cell cycle, with the extent of this decrease being dependent on the dosage [253]. This study represents the inaugural investigation into the impact of **36** on the induction of cell cycle arrest, with a specific focus on MDA-MB-231 breast cancer cells. Compound **28**, however, is not an inhibitor of amyloid beta-peptide (25–35) or Aβ_25–35_ [254]. This neurotoxin could induce oxidative damage in mitochondria, thus promoting neuronal cell death in patients with Alzheimer’s disease (AD). Compound **36** did not trigger any mitochondrial dysfunction or apoptotic induction in Aβ_25–35_-stimulated rat pheochromocytoma (PC12) cells [254].

### 7.6. Docosahexaenoic Acid

Marine algae are rich in polyunsaturated fatty acids (PUFAs), with omega-3 polyunsaturated fatty acids (ω-3 PUFAs) as their main constituent [255]. The ω-3 PUFAs are essential for regulating the structural features of immune and neural cellular membranes [256]. The two main forms of ω-3 polyunsaturated fatty acids (PUFAs) that are produced by marine algae are the long-chained 22:6n-3 docosahexaenoic acid (DHA) **37** (Figure 7) and 20:5n-3 eicosapentaenoic acid (EPA) **38** (Figure 7), as well as its precursor alpha-linolenic acid (ALA) **39** (Figure 7) [257]. The ω-3 PUFAs have anticancer properties that act against multiple carcinomas and can be used to supplement cancer treatments [258,259]. Apoptosis induction was successfully facilitated by compound **37** [259], and it was also seen in compound **38** at a slightly lower magnitude [260].

The inhibition of the PI3K/Akt pathway was the key contributor to **37**’s apoptotic processes without inflicting any damage to non-tumor cells [260]. Furthermore, compound **37** has been shown to upregulate the tumor suppressor oxidative stress-induced growth inhibitor 1 (OSGIN1), which may cause apoptosis in the breast cancer cell lines MCF-7 and Hs578T [255]. Compound **37** alters the G2/M phase of the cell cycle to prevent MDA-MB-231 breast cancer cells from proliferating [260]. The anti-invasive activity against MDA-MB-231 cancer cells was demonstrated by the upregulation of type II cytoskeletal 1 (KRT1) proteins and keratin [261]. In addition to these apoptotic processes, ferroptosis was induced, and drug-resistant breast cancer cells’ resistance was reversed by fish-derived **37** [262]. Compound **37** has been demonstrated to increase the amount of lipid peroxides in the cellular membrane, which could contribute to oxidative stress and apoptosis [262]. Furthermore, when coupled with Dox as a treatment for drug-resistant carcinomas, marine-derived **37** has demonstrated a synergistic anticancer effect [262]. Clinical trials investigated **37**’s anticancer potential in addition to chemotherapeutic drugs for patients with breast cancer. The ClinicalTrials.gov database contains a list of these clinical trials.

Only **37** of the previously listed omega-3 polyunsaturated fatty acids induced apoptosis and reduced gastric adenocarcinoma (AGS) cell growth without targeting non-tumoral gastric cells (GES-1 and HEK-293) [263]. At a lower dosage, compound **37** showed a considerable reduction in AGS cell growth that was comparable to the antitumor medication cisplatin [263]. According to these results, **37** may be used as an adjuvant in the treatment of gastric cancer in conjunction with chemotherapy medications such as cis-platin [263]. Compound **37** derived from marine algae is far from being the only anticancer resource available for different types of carcinomas.

## 8. Marine Cyanobacteria

### Apratoxins

Marine cyanobacteria are known to produce chemodiverse compounds with anticancer activities [264]. One group of compounds, known as apratoxins, has been found to have a significant impact on the apoptosis pathway, making them effective in fighting cancer. Moreover, several studies on the structure activity relationship of apratoxins were conducted to investigate their anticancer activities; hence, this section focuses on apratoxins.

**Figure 8:**
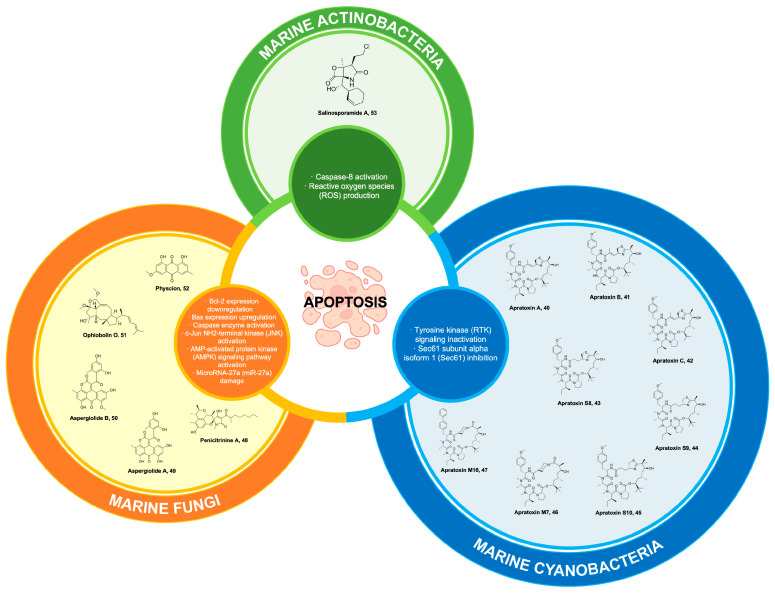
Marine natural products from cyanobacteria, fungi, and actinobacteria and their synthetic derivatives, with their mechanisms of action targeting the apoptosis pathways.

A cyclodepsipeptide compound, apratoxin A **40** (Figure 8), was derived from the marine cyanobacterium *Lyngbya majuscula* and found to demonstrate significant cytotoxicity with IC50 values of 0.36 nM and 0.52 nM against LoVo and KB cancer cells [265]. Compound **40** was regarded as the most potent among the various cyclodepsipeptide compounds isolated from the species by Luesch et al. in 2001. The investigation of structure-activity relationships (SAR) was conducted by synthesizing and evaluating analogues of apratoxin A, namely apratoxin B **41** (Figure 8) and C **42** (Figure 8), as well as *E*-dehydroapratoxin A. The results indicated varying degrees of reduced cytotoxicity, which were found to be strongly influenced by the structural characteristics of the compounds [266]. The substitution of the thiazoline ring in **40** with an oxazoline ring, which is more readily available, led to the production of an oxazoline derivative of **40**. This derivative exhibited a slightly lower but comparable level of cell proliferative activity against HeLa cells [267].

Through the utilization of functional genomics, **40** has exhibited its ability to induce cytotoxic effects on tumors. This is achieved by causing cell cycle arrest and promoting apoptosis. Furthermore, it has been observed that **40** exhibits interference with receptor tyrosine kinase (RTK) signaling, leading to the complete inhibition of phosphorylation of the transcription factor STAT3 [268]. Meanwhile, proteomics analysis was employed to ascertain the impact of **40** on N-glycosylation in relation to various receptor tyrosine kinases associated with cancer. This disruption ultimately results in the swift degradation of cancer cells through the proteasomal pathway [269]. Compound **40** has been found to inhibit the process of co-translational translocation of secretory and membrane proteins within the endoplasmic reticulum (ER), which is responsible for the synthesis of N-glycoproteins. Furthermore, **40** inhibits protein translocation by specifically binding to transport protein Sec61 subunit alpha isoform 1 (Sec61α), the primary component of the protein translocation channel. This discovery sheds light on the distinct effects of substrate-selective (contransin) [270] and substrate-nonselective (apratoxin) [271] Sec61 inhibitors on the process of secretory protein biogenesis [269]. The Sec61 protein complex functions as a crucial regulator for the translocation of nascent peptides from the cytosol into the endoplasmic reticulum (ER), both during and after translation. Furthermore, the observation was made that **40** interferes with the process of membrane integration at a stage that is likely common to all Sec61 substrates, occurring before the nascent chain docks onto the Sec61 lateral gate [269]. Compound **40** has been considered useful as a chemical probe to investigate human epidermal growth factor receptor (HER, ErbB) signaling in human breast and cancer malignancies that are resistant to treatment [272].

Another study examined **40**’s in vivo toxicity to determine if its biological activity and possible anticancer drugs were related [273]. The organs frequently affected by anticancer medications, including bone marrow, liver, gastrointestinal tract, and kidney, exhibited no observable signs of toxicity. Nevertheless, it was observed that the pancreatic tissue exhibited a significant affinity towards **40**. The potential toxicity of **40** may be linked to its propensity to accumulate in the normal pancreas, resulting in pancreatic atrophy. This observation suggests that **40** could have therapeutic implications for cancers characterized by active secretive pathways, such as pancreatic cancer [273].

In the study conducted by Cai et al. [274], efforts were made to enhance the therapeutic index of **40** by identifying compounds with robust and enduring in vitro and in vivo anticancer properties. This endeavor resulted in the identification of three novel apratoxins, namely apratoxins S4, S8, and S9. Apratoxin S8 **43** (Figure 8) has been found to exhibit the highest yield of macrocyclization [275], while apratoxin S9 **44** (Figure 8) has been identified as the most potent analogue against HCT116 [275]. To achieve a combination of balanced potency, stability, and synthetic yield, a novel hybrid compound called apratoxin S10 **45** (Figure 8) was designed [275]. Compound **45** demonstrated inhibitory effects on angiogenesis in an in vitro setting. It effectively suppressed the secretion of vascular endothelial growth factor A (VEGF-A) and interleukin 6 (IL-6) from cancer cells. These two molecules are well-established promoters of endothelial cell proliferation, migration, and the formation of blood vessels. Compound **45** suppresses several receptor tyrosine kinases (RTKs) to inhibit cancer cells from highly vascularized tumors and reduce angiogenesis [275].

Moreover, an investigation into the potential of **45** in combating pancreatic cancer was conducted. Compound **45** is considered one of the most promising candidates within the apratoxin family due to its high potency, stability, and ease of synthesis [276]. Moreover, **45** demonstrated inhibitory effects on various cytokines secreted by stromal cells, indicating its potential to suppress not only the secretion of cytokines by pancreatic cancer cells but also the levels of factors secreted by different types of cancer cells. The tissue distribution analysis of **45** revealed a significant concentration in pancreatic tissue within an orthotopic mouse model using patient-derived xenografts of pancreatic origin [276]. The results of this study demonstrated the anti-tumor efficacy of apratoxin **45** in a pancreatic cancer model, which was attributed to its antiproliferative properties.

The positive discoveries pertaining to the fundamental structural characteristics and alterations of **40** have subsequently facilitated the development and identification of its molecular target, as well as the enhancement of its biological and cytotoxic efficacy. Therefore, an alternative methodology referred to as apratoxin mimetics was developed in order to synthesize and investigate the cytotoxic properties of apratoxin derivatives that possess analogous conformations to **40** [277]. In the initial iteration, the modified cysteine residue (moCys) moiety was substituted with a series of seven basic amino acids (Apratoxin M1-M7). These amino acids were synthesized through the utilization of solid-phase peptide synthesis and solution-phase macrolactamization techniques [277]. Apratoxin M7 **46** (Figure 8), which incorporates a piperidinecarboxylic acid moiety, demonstrated significant cytotoxic activity against HCT116 cells with an inhibitory concentration (IC50) of 120 nM. In the subsequent generation, the amino acid residue substitution was performed on the tripeptide Tyr(Me)–MeAla–MeIle moiety in **46**. This led to the creation of apratoxin M16 **47** (Figure 8), which exhibited significantly enhanced potency (IC50 = 1.1 nM) [277]. Compound **47** featured the substitution of Tyr(Me) with biphenylalanine (Bph). Notably, apratoxin **47** demonstrated greater potency against HCT116 cells compared to **40** (IC50 = 2.8 nM). The design and synthesis of **40** mimetics based on conformational principles have resulted in notable modifications to the ring component’s structure while still maintaining a remarkably high level of cytotoxicity against cancer cells.

## 9. Marine Fungi

### 9.1. Penicitrinine A

Penicitrinine A, **48** (Figure 8), represents a newly discovered alkaloid possessing a distinctive spiro skeleton. Compound **48** was extracted from the marine-derived fungus known as *Penicillium citrinum* [278]. It exhibited antiproliferative efficacy against various tumor types, encompassing stomach cancer, lung cancer, colon cancer, oral epidermoid carcinoma, liver cancer, nasopharynx cancer, esophagus cancer, breast cancer, lung cancer, and the A-375 human malignant melanoma cell line [278]. Compound **48** has been observed to exert a notable apoptotic effect on A-375 melanoma cancer cells through the downregulation of Bcl-2 expression and the upregulation of Bax expression. Furthermore, an assessment was conducted to investigate the antimetastatic properties of **48** in A-375 cells. The findings demonstrated that **48** exerted a notable inhibitory effect on the metastatic behavior of A-375 cells through the modulation of MMP-9 expression and its corresponding inhibitor, tissue inhibitor matrix metalloproteinase 1 (TIMP-1). The findings of this study indicated that **48** could be a potential chemotherapeutic agent for the treatment of melanoma A-375 cells [278]. Despite these promising studies elucidating the anticancer potential of **48**, it is important to note that no preclinical trials have been conducted recently. Nonetheless, further investigations and preclinical trials are important to harness the full therapeutic potential of this compound and advance its candidacy as a promising chemotherapeutic agent for melanoma treatment.

### 9.2. Aspergiolide 

Aspergiolide A **49** belongs to the class of anthraquinone derivatives and possesses a unique chemical structure known as the naphtho[1,2,3-de]chromene-2,7-dione skeleton (Figure 8). Compound **49** is derived from the marine fungus *Aspergillus glaucus*, which was obtained from the marine sediment surrounding the mangrove roots found in the Fujian Province of the People’s Republic of China. In a study conducted by Wang et al. (2014) [279], it was demonstrated that **49** exhibits significant inhibitory effects on topoisomerase II. The results of the in vitro investigation demonstrated that **49** exhibits inhibitory effects on the proliferation of diverse human cancer cells. Furthermore, it was observed that **49** induces apoptosis, specifically in hepatocellular carcinoma BEL cells. The cytotoxic activities of analogues of **49** were also assessed. Aspergiolide B **50** (Figure 8) exhibited significant cytotoxic effects on the HL-60 and A-549 cell lines, as evidenced by IC50 values of 0.51 and 0.24 µM, respectively. These findings suggest that the *O*-methylation of OH-8 did not exert any detrimental influence on its bioactivities [280]. In a preliminary investigation on drug development carried out by Wang et al. (2014), it was observed that the maximum tolerable dose exceeded 400 mg/kg [279]. Furthermore, **50** was found to lack genotoxic or cardiotoxic potential, as evidenced by the absence of noteworthy elevations in micronucleus rate or inhibitions of the hERG channel. The uptake and transport assay conducted in monolayer Caco-2 cells demonstrated that **50** exhibited absorptions via the active transport pathway. The findings of this study suggest that **50** exhibits anticancer properties by selectively inhibiting topoisomerase II, employing a structural and mechanistic resemblance to adriamycin while displaying significantly reduced toxicity [280].

In 2017, a novel derivative of **49**, AS1041, was synthesized and reported to exhibit cytotoxic effects across a wide range of human cancer cell lines. AS1041 showed half maximal inhibitory concentrations (IC50) ranging from 1.56 to 10.30 µM against chronic myelogenous leukemia cells (K562), promyelocytic leukemia cells (HL-60), acute T lymphocytic leukemia cells (Kasumi-1), T lymphocytic leukemia (Jukrat cells), cervical carcinoma (HeLa cells), cervical cancer cell lines CaSki, hepatocellular carcinoma (BEL-7402), lung cancer cells (A549), breast cancer cells (MDA-MB-231), and prostate cancer cells (PC-3), with K562 cells being the most sensitive to AS1041. Subsequent experiments revealed that AS1041 inhibited the proliferation of K562 in a concentration- and time-dependent manner, induced cell cycle arrest at the G2/M checkpoint, and induced a non-caspase-dependent apoptosis [281].

A study on the derivative of **49**, AS1041, found that it induces cell antiproliferation and senescence in K562. The senescence activity of AS1041 against K562 was contributed by the activation of senescence-promoting proteins P53/P21 and P16^INK4a^/Rb pathways via an increase in ROS generation and the degradation of BCR-ABL genes via the ubiquitin proteosome system (UPS). The apoptosis assay revealed that AS1041 (12.5 µM) slightly induced cell apoptosis of about 16% at the early stage and 37% at the late apoptosis stage [282]. This investigation suggests that AS1041 could be exploited for a one-two punch therapeutic approach in treating chronic myelogenous leukemia. AS1041 will act as the first drug that induces vulnerability (e.g., senescence) in the cancer cells that are exploited by the second drug [283]. Moreover, the AS1041 monotherapy displayed noteworthy clinical benefits, such as a decrease in the risk of drug–drug interactions due to its dual role to induce senescence and apoptosis [282].

### 9.3. Ophiobolin O

Ophiobolin O **51** (Figure 8) is a sesterterpene compound that has been extracted from a marine fungus known as *Aspergillus ustus*. Compound **51** has demonstrated promising potential as an antineoplastic agent for the treatment of human breast carcinoma. In addition, compound **51** exhibited a decline in the survival rate of MCF-7 cells, a type of human breast cancer cell, in a manner that was dependent on both the duration of exposure and the concentration of **51**. Furthermore, **51** effectively triggered apoptosis in MCF-7 cells [284]. The inhibitory effect of **51** on cell cycle progression at the G0/G1 phase has been elucidated. The induction of Bcl-2 phosphorylation by **51** was observed in MCF-7 cells, leading to the promotion of apoptosis. This effect was mediated through the activation of c-Jun NH2-terminal kinase (JNK) [284]. The results of this study have garnered attention due to the observed deficiency in caspase-3 activity, a crucial component of the apoptosis process, in MCF-7 breast cancer cells [285]. The study conducted by Lv et al. (2015) provided additional evidence supporting the occurrence of G1 phase arrest in human breast cancer MCF-7 cells because of treatments with **51** [286]. The researchers also observed a decrease in the phosphorylation levels of AKT and GSK3β, as well as a downregulation of cyclin D1 expression following exposure to **51**. Compound **51** was subjected to in vivo experimentation to evaluate its effects on tumor growth suppression and toxicity in mouse xenograft models [286]. In addition, **51** reversed the resistance of MCF/ADR cells to adriamycin (ADM) [287], a commonly used drug in breast cancer treatment [288]. The combination effect of **51** (0.1 µM) and ADM (3 µM, 6 µM, or 9 µM) reversed the ADM resistance of MCF/ADR by downregulating the expression of the resistance genes, which increases the intracellular accumulation of ADM and subsequently triggered the G2/M phase arrest and ADM-induced apoptosis [288]. The findings presented in this study provide substantial evidence that **51** exhibits significant potential as a promising pharmaceutical agent for the treatment of breast cancer.

### 9.4. Physcion

Marine fungi exhibit a remarkable capacity to produce a diverse array of distinct secondary metabolites, which possess potent anticancer properties [289]. Physcion **52** (Figure 8) has been obtained from a marine fungus *Microsporum* sp. [290]. Compound **52** belongs to the anthraquinone class and has been scientifically established to possess the ability to initiate apoptosis, specifically in cancerous cells. The study conducted by Wijesekara et al. (2014) elucidated the capacity of **52** to elicit apoptosis by means of downregulating the expression of Bcl-2, upregulating the expression of Bax, and activating the caspase-3 pathway. Furthermore, the investigation elucidated the mechanism by which **52** elicits the generation of reactive oxygen species (ROS) within HeLa cells [290].

Compound **52** has been reported to exhibit antitumorigenic properties on a diverse range of carcinoma cells, primarily by impeding cellular proliferation, inducing apoptosis, and causing cell cycle arrest [291]. The viability of gastric cancer cell line (SGC-7901) cells was diminished by **52** in a manner that was dependent on the dosage and duration of exposure. This reduction in viability was observed to activate the mitochondrial/caspase apoptotic pathway, as evidenced by the decline in mitochondrial membrane potential and the release of cytochrome. In human gastric cancer cells, the induction of apoptosis was observed through the activation of the AMP-activated protein kinase (AMPK) signaling pathway by **52** [291]. This process necessitated the generation of reactive oxygen species (ROS) and the subsequent activation of AMPK [292]. Chen et al. (2015) conducted a study to investigate the relationship between the modulation of **52**’s effect on extracellular matrix metalloproteinase inducer (EMMPRIN) and AMPK/hypoxia-inducible factor 1α (HIF-1α) in colorectal cancer cell lines (CRC) HCT116 [293]. The viability of tumor cells was found to be inhibited by **52** in a manner that was dependent on both the dosage and duration of treatment. The study revealed that **52** elicits mitochondrial apoptosis through the downregulation of the AMPK/HIF-1α signaling pathway [293]. In a study conducted by Pang et al. (2016), it was observed that the application of **52** at varying concentrations (5, 10, and 20 µmol/L) resulted in a dose-dependent reduction in cell viability and colony formation in the CNE2 cell line, which is associated with nasopharyngeal carcinoma. Compound **52**, when administered at concentrations of 10 and 20 µmol/L, exhibited a dose-dependent inhibitory effect on the progression of the cell cycle, specifically at the G1 phase [292]. Additionally, it triggered both caspase-dependent apoptosis and autophagy in CNE2 cells. Moreover, the administration of **52** resulted in the excessive production of reactive oxygen species (ROS) in CNE2 cells. This led to the disruption of the microRNA-27a/zinc finger and BTB domain containing the 10 gene (miR-27a/ZBTB10) axis, which subsequently caused the repression of the transcription factor Sp1. It is worth noting that Sp1 played a crucial role in the physcion-induced processes of apoptosis and autophagy [294]. Compound **52** has been found to induce apoptosis in hepatocellular carcinoma cell lines by upregulating miR-370 through the AMPK/Sp1/DNA methyltransferase 1 (DNMT1) signaling pathway [292]. In a study conducted by Gao et al. (2017) using in vitro techniques, the researchers demonstrated the inhibitory effects of **52** on cell proliferation, apoptotic induction, and cell cycle arrest in acute lymphoblastic leukemia (ALL) cell lines. The administration of **52** resulted in the downregulation of homeobox 5 gene (HOXA5) expression, a key factor contributing to its antileukemia properties [295]. Further investigations showed that **52** inhibits the proliferation of human prostate cancer (PC3) cells in a dose-dependent manner. Compound **52** also induces apoptosis on PC3 via the Ras/Bcl-2 family signaling pathway by downregulating the antiapoptotic proteins Ras, Bcl-xL, and Bcl-2 and upregulating caspase-3, caspase-8, and caspase-9 [296].

Recently, compound **52** has been shown to improve sorafenib’s therapeutic efficacy against hepatocellular carcinoma (HCC), which is resistant to sorafenib. Combining the treatment using **52** with sorafenib resulted in the induction of apoptosis in HepG2-SR (sorafenib-resistant hepatoma cells) and Huh7-SR (sorafenib-resistant Huh-7 cells) via the overexpression of Bax and downregulation of Bcl-2 expression, as well as an increase in cleaved caspase-3 and poly (ADP—ribose) polymerase. Furthermore, **52** downregulated miR-370 inhibitors to lower the expression of the glycolysis regulator PIM1, which enhanced the sensitivity of sorafenib to suppress glycolysis in sorafenib-resistant HCC cells [297]. Additionally, studies were conducted to determine how to improve the solubility of **52** by generating its nanoparticles (NPs) for oral bioavailability with the potential for antioxidant and anticancer effects. When compared to the unprocessed **52** (A549: IC50 = 9.9 µg/mL; HepG2: IC50 = 7.85 µg/mL; MDA-MB-231: IC50 = 7.45 µg/mL), the **52** NPs demonstrated significant cytotoxicity against the cancer cell lines A549 (IC50 = 4.12 µg/mL), HepG2 (IC50 = 2.84 µg/mL), and MDA-MB-231 (IC50 = 2.97 µg/mL). The solubility and dissolving rate of **52** were enhanced by the reduction of particle size, wh.ich also enhanced its cytotoxic effect. This could lead to the creation of new antiproliferative formulations [298]. Despite demonstrating promising results in preclinical trials conducted on various carcinoma cells, compound **52** has not been documented in the National Institutes of Health (NIH) database as having undergone any clinical trials for potential use in anticancer therapeutics. The cytotoxicity potential of **52** toward human cells remains to be ascertained.

## 10. Marine Actinobacteria

### Salinosporamide A

Salinosporamide A, **53** (Figure 8) exhibits significant potential as a pharmaceutical agent. Compound **53** was derived from the obligate marine actinomycetes *Salinospora tropica* [299]. The compound’s distinctive gamma-lactam-beta-lactone bicyclic ring structure and its strong efficacy against the 20S proteasome, a biologically significant target in cancer therapy [300], have attracted interest in the quest for a more powerful anticancer agent capable of overcoming the intrinsic resistance of cancer cells to bortezomib (an FDA-approved proteasome inhibitor) [301]. Compound **53** was developed into a drug named Marizomib^®^. One notable distinguishing feature of Marizomib^®^, in comparison to other proteasome inhibitors, is its capacity to traverse the blood–brain barrier and effectively impede proteasome activity within the brain. Preclinical investigations have additionally exhibited the presence of antitumor properties in cranial glioma xenograft models [302]. The induction of apoptosis is initiated by Marizomib^®^ through a mechanism that relies on caspase-8 and the production of reactive oxygen species [303]. A comprehensive preclinical assessment has been conducted to determine the efficacy of Marizomib^®^ as a standalone treatment and in combination with other therapies for various hematologic malignancies (such as multiple myeloma and leukemia) as well as solid tumors (including prostate and pancreatic cancer cells). These evaluations have laid the foundation for the ongoing clinical trials [304,305,306]. In the year 2014, the U.S. Food and Drug Administration bestowed orphan drug designation upon Marizomib^®^ for its potential application in the treatment of multiple myeloma [307].

Eight clinical trials involving Marizomib^®^ have been reported in the National Institute of Health database. Twenty percent of the Phase I trials are still in progress, but 80% have concluded successfully. Patients with solid tumors and advanced cancers have successfully completed the Phase I clinical study of Marizomib^®^. An 11% response rate was obtained from the trial, and the adverse effects that were noted included headache, fatigue, nausea, dizziness, diarrhea, and vomiting. When Marizomib^®^ doses are administered, peripheral neuropathy and hematologic toxicity do not occur as frequently as they have been reported with alternative proteasome inhibitors [308]. Prior research has demonstrated that the combined use of proteasome inhibitors and HDAC inhibition exhibits a synergistic effect in suppressing tumor growth. This effect is attributed to the upregulation of pro-apoptotic proteins, the downregulation of antiapoptotic proteins, and the induction of reactive oxygen species (ROS), which in turn promote DNA damage [303,309,310]. This study assessed the effectiveness of a Phase I clinical trial examining the combination of the histone deacetylase inhibitor (HDAC) vorinostat with the proteasome inhibitor Marizomib^®^ in patients with non-small cell lung cancer, pancreatic cancer, melanoma, or lymphoma. The evaluation covered several areas, such as the treatment’s safety profile, early antitumor activity, and pharmacokinetics and pharmacodynamics. The study’s conclusions showed that the treatment’s concurrent administration had no appreciable effect on pharmacokinetics (PK) or pharmacodynamics (PD), nor did it raise toxicity levels in any way. Sixty-one percent of the patients showed no change in their health, while the remaining 39% had their tumor measurement decrease. Combining vorinostat and Marizomib^®^ proved to be a feasible treatment option; the most common side effects described were exhaustion, nausea, vomiting, constipation, and diarrhea [311]. A further clinical trial was initiated to assess the effects of Marizomib^®^, pomalidomide, and low-dose dexamethasone in patients with relapsed and refractory multiple myeloma (RRMM). Finding the maximum tolerated dosage (MTD) and suggesting the Phase II dose of Marizomib^®^ for patients diagnosed with relapsed and refractory multiple myeloma (RRMM) are the goals of this study. A total response rate of 53% and a clinical benefit rate of 64% were found. The results of this study showed that 0.5 mg/m^2^ was the recommended Phase II dose (RP2D) of Marizomib^®^. Moreover, no cases of dose-limiting toxicities were noted during the investigation [312].

The significant findings made it easier for the Phase II clinical trial, including Marizomib^®^, to proceed and be successfully completed in patients with relapsed or refractory multiple myeloma. There have been significant developments in the use of proteasome inhibitors, such as Marizomib^®^, in the management of hematological malignancies within the recent year. However, research on solid malignancies indicates that these inhibitors’ effectiveness in treating solid tumors is still being studied [313,314]. The Phase I/II clinical trial explored the possible synergistic effect of Marizomib^®^ with bevacizumab in the treatment of glioblastoma. Marizomib^®^ crosses the blood–brain barrier (BBB), which makes it a possible treatment for patients with central nervous system (CNS) malignancies like glioblastoma (GBM) [315]. This contrasts with other therapeutic proteosome inhibitors like bortezomib and carfilzomib. Because proteosome inhibition causes glioma cells to produce vascular endothelial growth factors (VEGF), bevacizumab and Marizomib^®^ were combined because of this [316]. The highest dose of Marizomib^®^ in patients with GMB was 0.8 mg/m^2^ when used as monotherapy or in combination with bevacizumab, according to the safety profile. Nevertheless, patients with recurrent GBM did not show appreciable improvement when Marizomib^®^ (0.8 mg/m^2^) was used in combination with bevacizumab (10 mg/kg), which is the current medication of choice for treating GBM [315].

Numerous studies also showed that Marizomib^®^ could be used in conjunction with other forms of therapy. The combination treatment with the latest generation TRAIL (tumor necrosis factor-related apoptosis ligand) receptor agonist, IZI1551, synergistically induced apoptosis via a caspase-dependent pathway in patient-derived GBM. Treatment with IZI1551 plus Marizomib^®^ exposed cells to nuclear and cellular morphologies, including karyopyknosis and blebbing. Additionally, cells were absent when Q-VD-Oph, a pan-caspase inhibitor, was present, suggesting that apoptosis was the cause of the cell death [317]. Combined treatment with cisplatin in cervical cancer also exhibited synergistic effects to induce cell growth and apoptosis in in vitro and in vivo tests. Marizomib^®^ increases the expression of the Angiopoietin 1 (Ang-1) gene, inhibits the expression of stem cell factor (SCF) and FMS-like tyrosine-3 kinase (FLT-3), and induces caspase-3 cleavage and poly (ADP-ribose) polymerase (PARP) to enhance the cytotoxic effect and apoptosis produced by cisplatin. The potential of Marizomib^®^ as an adjuvant to current medications in combination therapy for patients with cervical cancer was established by the synergistic impact of Marizomib^®^ plus cisplatin [318]. Furthermore, by impairing the potential of the mitochondrial membrane and increasing the generation of reactive oxygen species (ROS), a co-treatment of Marizomib^®^ with panobinostat, an oral pan-histone deacetylase inhibitor approved by the FDA for the treatment of cancer [318], induces apoptosis and mitochondrial dysfunction in both pediatric (pHGG) and adult high-grade gliomas (aHGG). Nevertheless, resistance was noted to this combination at clinically relevant doses, which restricted the co-treatment’s efficacy as a therapeutic agent. Subsequent investigation revealed that the highly integrated tricarboxylic (TCA) cycle of Marizomib^®^ with panobinostat, which upregulated glycolysis and activated the energy metabolism to replenish the supply of ATP for cell growth and survival, was responsible for the observed resistance to the drug in glioma-resistant cells. These findings call for more research on treating glioma-resistant cells by focusing on metabolic vulnerability [319].

The ongoing Phase III clinical trial involving Marizomib^®^ in combination with tomozolomide-based radiochemotherapy for newly diagnosed glioblastoma has thus far shown no significant improvement in overall survival or progression-free survival compared to the control group, as documented by the National Institute of Health’s clinical trials database (https://classic.clinicaltrials.gov/ct2/show/NCT03345095?term=NCT03345095&draw=2&rank=1 (accessed on 20 November 2023)). The comparable median survival rates between the two groups indicate that the addition of Marizomib^®^ did not enhance the outcomes in this patient population. It is important to note that secondary analyses, particularly investigating the impact of *O*-6-methylguanine-DNA-methyltransferase promoter methylation on Marizomib^®^ efficacy and other relevant endpoint treatments, are still in progress and have not been published. These analyses are anticipated to provide deeper insights into the specific factors influencing Marizomib’s effectiveness and may shed light on potential avenues for optimizing its therapeutic benefits in the treatment of glioblastoma [320].

## 11. Future Perspective and Conclusions

Marine organisms and microorganisms serve as an extensive source of natural products that exhibit significant cytotoxic and antiproliferative properties. Significantly, most marine-derived molecules that have obtained approval as pharmaceutical agents or are currently undergoing clinical trials exhibit anticancer properties. This is achieved through the selective modulation of various vital cellular mechanisms, including DNA replication, regulation of the cell cycle, and maintenance of the cytoplasmic membrane’s integrity. The thriving ecosystem highlights the promising opportunity for an expedited growth of pharmaceutical advancements derived from marine sources, presenting a novel frontier in the therapy of various forms of cancer.

In this review, we have elucidated various pivotal directions and potential pathways for the future utilization of marine-derived anticancer compounds and their synthetic derivatives in their progression towards clinical applications. Primarily, the optimization of combination therapies involving established anticancer drugs represents a compelling area of study. By harnessing the combined potential of marine-derived compounds and conventional treatments, we can optimize therapeutic effectiveness while minimizing adverse effects, thereby leading to enhanced patient outcomes.

Further exploration into the complex molecular mechanisms that govern the activities of compounds derived from marine sources continues to be a key focus for future scientific inquiries. Deciphering the complex mechanisms by which cancer cells interact with each other, as well as the underlying genetic processes involved, holds the potential to enhance our comprehension and inform the advancement of meticulously tailored therapeutic approaches. Furthermore, the investigation of novel molecular targets has the potential to reveal previously unknown pathways for the treatment of cancer, thereby expanding the range of available therapeutic choices.

The synthesis of the derivatives obtained from natural marine products has already demonstrated their efficacy as anticancer agents. Future investigations will probably prioritize the optimization and expansion of the synthesis procedures, thereby enhancing the availability of these compounds for clinical utilization. The formidable challenge of drug resistance in cancer treatment necessitates the development of innovative strategies. Future investigations may focus on the development of methodologies to address resistance, potentially through the alteration of marine-derived compounds or their strategic integration with other agents. The elucidation of the complex molecular interactions between these marine compounds and cancer cells provides a means to optimize their efficacy. This line of inquiry is expected to result in the creation of compounds that exhibit improved accuracy and diminished off-target impacts. Concurrently, the advancement of sophisticated pharmaceutical delivery systems will continue to be a primary area of emphasis in the forthcoming scientific investigations. Cutting-edge scientific advancements, such as nanotechnology and nanoparticles, exhibit the potential to transport therapeutic payloads with precision to malignant cells while minimizing damage to normal tissues.

Finally, given the restricted availability of these compounds in nature, which can only be found in certain organisms that cannot be cultivated, it is crucial to develop reliable methods for screening and studying the pharmacological properties of these identified compounds. Gaining insights into the complex structures and underlying mechanisms of these valuable anticancer agents will facilitate their potential replication within controlled laboratory settings. This, in turn, will establish a foundation for the development of a sustainable and reliable source of these highly sought-after therapeutic compounds.

In conclusion, the outlook for research in this domain is primed for notable advancements, as marine-derived natural compounds are positioned to play a crucial role in the advancement of groundbreaking cancer therapies and treatment approaches. The vast and limitless possibilities presented by the ocean’s resources provide unprecedented opportunities for advancing the understanding and treatment of cancer, as well as enhancing the wellbeing of numerous individuals on a global scale.
